# Antimalarial Plants Used across Kenyan Communities

**DOI:** 10.1155/2020/4538602

**Published:** 2020-06-12

**Authors:** Timothy Omara

**Affiliations:** ^1^Africa Centre of Excellence II in Phytochemicals, Textiles and Renewable Energy (ACE II PTRE), Moi University, Uasin Gishu County, P.O. Box 3900-30100, Eldoret, Kenya; ^2^Department of Chemistry and Biochemistry, School of Sciences and Aerospace Studies, Moi University, Uasin Gishu County, P. O. Box 3900-30100, Eldoret, Kenya; ^3^Department of Quality Control and Quality Assurance, Product Development Directory, AgroWays Uganda Limited, Plot 34-60 Kyabazinga Way, P.O. Box 1924, Jinja, Uganda

## Abstract

Malaria is one of the serious health problems in Africa, Asia, and Latin America. Its treatment has been met with chronic failure due to pathogenic resistance to the currently available drugs. This review attempts to compile phytotherapeutical information on antimalarial plants in Kenya based on electronic data. A comprehensive web search was conducted in multidisciplinary databases, and a total of 286 plant species from 75 families, distributed among 192 genera, were retrieved. Globally, about 139 (48.6%) of the species have been investigated for antiplasmodial (18%) or antimalarial activities (97.1%) with promising results. However, there is no record on the antimalarial activity of about 51.4% of the species used although they could be potential sources of antimalarial remedies. Analysis of ethnomedicinal recipes indicated that mainly leaves (27.7%) and roots (19.4%) of shrubs (33.2%), trees (30.1%), and herbs (29.7%) are used for preparation of antimalarial decoctions (70.5%) and infusions (5.4%) in Kenya. The study highlighted a rich diversity of indigenous antimalarial plants with equally divergent herbal remedy preparation and use pattern. Further research is required to validate the therapeutic potential of antimalarial compounds from the unstudied claimed species. Although some species were investigated for their antimalarial efficacies, their toxicity and safety aspects need to be further investigated.

## 1. Introduction

Globally, malaria continues to be in the top list of the major global health challenges. A global estimate of 655,000 malarial deaths was reported in 2010 of which 91% were in Africa and 86% of these were children under 5 years of age [1, 2]. Africa is particularly more susceptible, and conservative estimates cited that malaria causes up to 2 million deaths annually in Africa [3, 4]. The World Health Organization reported that about 2 billion people in over 100 countries are exposed to malaria, and the situation is exacerbated on the African continent which is characterized by limited access to health services and chronic poverty [5]. In East Africa and Kenya particularly, malaria remains endemic in the Lake Victoria basin and the coast with the country's highest rate of infection at 27% (6 million cases) in 2015 from 38% in 2010 [6, 7]. The Kenyan population at risk of malaria as of 2016 was estimated at 100% [5]. *Anopheles gambiae* and *A. funestus* are the primary vectors of malaria in East Africa [8], while *Plasmodium falciparum* and *P. vivax* are the deadliest malarial parasites in sub-Saharan Africa.

The misuse of chloroquine in the management of malaria has led to the development of chloroquine-resistant parasites worldwide [9]. In Kenya, the use of chloroquine has been discontinued as the first line treatment for malaria due to the prevalence of resistant *P. falciparum* strains [10, 11]. Artemisinin-based combination therapy (ACT) is currently the only available treatment option for malaria as the quinolines (quinine, chloroquine, and mefloquine) have been reported to cause cardiotoxicity, and the malarial parasites have already developed sturdy resistance to them [12, 13]. Unfortunately, resistance of *P. falciparum* to artemisinin has also been reported elsewhere [14].

The Kenyan government has attempted to reduce malaria incidences in Kenya through several approaches including entomologic monitoring, insecticide resistance management, encouraging the population to sleep under insecticide-treated mosquito nets, intermittent preventive treatment for pregnant women, and indoor residual spraying [6, 7, 15, 16]. The situation has been made more complicated by the emergence of pyrethroid-resistant mosquitoes throughout Western Kenya which prompted the government to declare no spraying of mosquitoes between 2013 and 2016 [6].

Malaria may manifest with relatively simple symptoms such as nausea, headache, fatigue, muscle ache, abdominal discomfort, and sweating usually accompanied by high fever [17]. However, at advanced stages, it can result in serious complications such as kidney failure, pulmonary oedema, brain tissue injury, severe anaemia, and skin discoloration [5, 18]. Conventional treatment is usually costly, and in rural Kenya just like in other parts of the world, the use of plants for either preventing or treating malaria is a common practice [3]. The current study attempted to gather comprehensive ethnobotanical information on various antimalarial plants and their use in Kenyan communities to identify which plants require further evaluation for their efficacy and safety in malaria management.

## 2. Methods

### 2.1. Literature Search Strategy and Inclusion and Exclusion Criteria

Relevant literature pertaining to antimalarial plants and their use in management of malaria and malarial symptoms in Kenya were sourced from Scopus, Web of Science Core Collection, PubMed, Science Direct, Google Scholar, and Scientific Electronic Library Online from November 2019 to February 2020 following procedures previously used [19–21]. The searches were performed independently in all the databases. Key search words such as malaria, vegetal, traditional medicine, ethnobotany, alternative medicine, ethnopharmacology, antimalarial, quinine, chloroquine, antimalarial activity, antiplasmodial activity, malaria management, and Kenya were used. All publishing years were considered, and reports with information on antimalarial or medicinal plants in Kenya were carefully screened. Thus, references contained within the returned scientometric results were assessed concerning their inclusion in the study, and further searches were carried out at the Google search engine using more general search terms, to broaden the search, as follows: words: malaria, plants, plant extract, vegetal, vegetal species, vegetal extract, traditional medicine, alternative medicine, complementary therapy, natural medicine, ethnopharmacology, ethnobotany, herbal medicine, herb, herbs, decoction, infusion, macerate, concoction, malaria fever, malaria incidence, and Kenya were used. The last search was done on 15^th^ February 2020. The search outputs were saved wherever possible on databases, and the author received notification of any new searches meeting the search criteria from Science Direct, Scopus, and Google scholar. For this study, only full-text original research articles published in peer-reviewed journals, books, theses, dissertations, patents, and reports on antimalarial plants or malaria phytotherapy in Kenya written in English and dated until February 2020 were considered.

Missing information in some studies particularly the local names, growth habit of the plants, and misspelled botanical names were retrieved from botanical databases: The Plant List, International Plant Names Index, NCBI taxonomy browser and Tropicos, and the Google search engine. Where a given species was considered as distinct species in different reports, the nomenclature as per the botanical databases took precedence. The traditional perception of malaria as well as the families, local names (Digo, Giriama, Kamba, Kikuyu, Kipsigis, Kuria, Luo, Markweta, Maasai, Nandi, and Swahili), growth habit, part (s) used, preparation, and administration mode of the different antimalarial plants were captured.

### 2.2. Data Analysis

All data were entered into Microsoft Excel 365 (Microsoft Corporation, USA). Descriptive statistical methods, percentages, and frequencies were used to analyze ethnobotanical data on reported medicinal plants and associated indigenous knowledge. The results were subsequently presented as tables and charts.

## 3. Results and Discussion

### 3.1. Antimalarial Plants Used in Kenya

**Figure 1:**
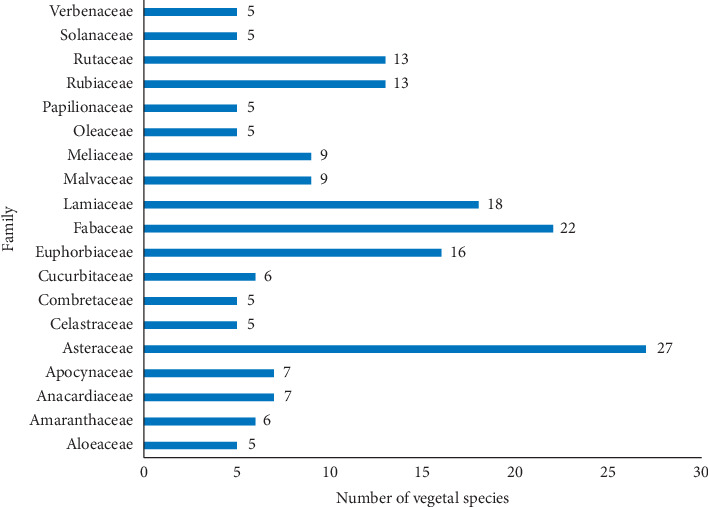
Major botanical families from which antimalarial remedies are obtained in Kenya.

In aggregate, 61 studies and reports identified 286 plant species from different regions of Kenya belonging to 75 botanical families distributed among 192 genera (Table 1). Asteraceae (36.5%), Fabaceae (29.7%), Lamiaceae (24.3%), Euphorbiaceae (21.6%), Rutaceae (17.6%), and Rubiaceae (17.6%) were the most common plant families (Figure 1). The most frequently encountered species were *Toddalia asiatica* (L.) Lam (11 times), *Aloe secundiflora* Engl. (10 times), *Azadirachta indica* A. Juss, *Carissa edulis* (Forsk.) Vahl., *Harrisonia abyssinica* Olive (9 times each), *Zanthoxylum chalybeum* Engl. (8 times), *Ajuga remota* Benth., *Rotheca myricoides* (Hochst.) Steane and Mabb, *Warburgia ugandensis* Sprague (7 times each), *Albizia gummifera* (J. F. Gmel.), *Erythrina abyssinica* Lam. ex DC., *Plectranthus barbatus* Andrews, *Rhamnus prinoides* L.'Herit, *Senna didymobotrya* (Fresen) Irwin and Barneby, and *Solanum incanum* L. (6 times). One botanically unidentified plant (*Ima*) was reported by Kuria et al. [11]. Decoction of a whole lichenized fungi (*Usnea* species and *Intanasoito* in Maasai dialect) and *Engleromyces goetzei* P. Henn. fungi were also reported to be used in management of malaria in rural Kenya [22, 23].

Some of the plants such as *Acacia mellifera* has been reported for treatment of malaria in Somalia [24], *Albizia coriaria* Welw. ex Oliver, *Artemisia annua* L., *Momordica foetida* Schumach*, Carica papaya* L., and *Catharanthus roseus* (L.) G. Don in Uganda [25, 26], Cameroon [27], and Zimbabwe [28], *Clematis brachiata* and *Harrisonia abyssinica* Oliv in Tanzania [29] and South Africa [30], *Artemisia afra* in Ethiopia [31], and *Tamarindus indica* L., *Carica papaya* L., and *Ocimum basilicum* L. in Indonesia [32].

### 3.2. Growth Habit, Part(s) Used, Preparation, and Administration of Antimalarial Plants

**Figure 2:**
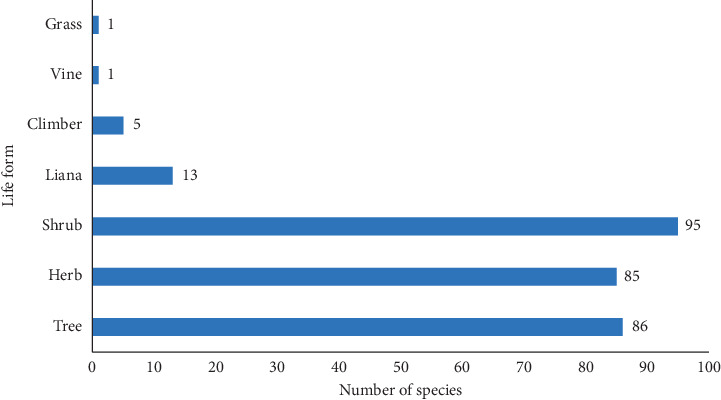
Growth habit of antimalarial plants used in Kenyan communities as per ethnobotanical surveys.

**Figure 3:**
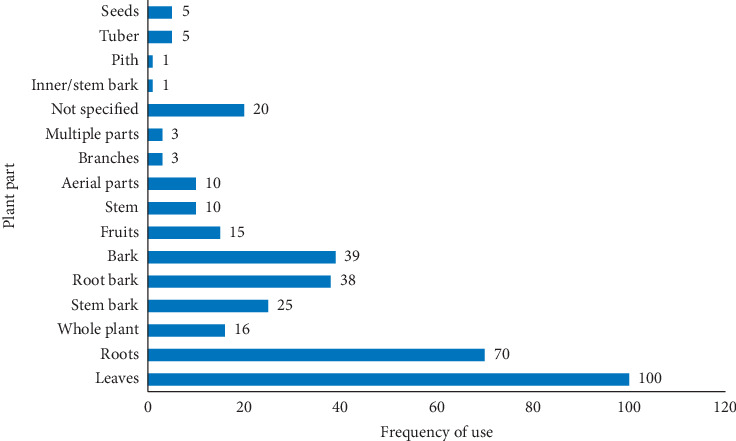
Frequency of the reported plant parts used for preparation of antimalarial remedies in Kenya.

Antimalarial plants used in Kenya are majorly shrubs (33.2%), trees (30.1%), and herbs (29.7%) (Figure 2), and the commonly used plant parts are leaves (27.7%) and roots (19.4%) followed by bark (10.8%), root bark (10.5%), and stem bark (6.9%) (Figure 3). Comparatively, plant parts such as fruits, seeds, buds, bulbs, and flowers which have reputation for accumulating phytochemicals are rarely used, similar to reports from other countries [26, 28, 33].

The dominant use of leaves presents little threat to the survival of medicinal plants. This encourages frequent and safe utilization of the plants for herbal preparations. Roots and root structures such as tubers and rhizomes are rich sources of potent bioactive chemical compounds [33], but their frequent use in antimalarial preparations may threaten the survival of the plant species used. For example, *Zanthoxylum chalybeum* and African wild olive (*Olea europaea*) have been reported to be threatened due to improper harvesting methods [2]. Thus, proper harvesting strategies and conservation measures are inevitable if sustainable utilization of such medicinal plants are to be realized.

Antimalarial remedies in Kenya are prepared by different methods. These include decoctions (70.5%), infusions (5.4%), ointments and steaming (1.3%), and roasting (0.3%). Preparation of antimalarial remedies from dry parts of one plant or several plants and ashes by using grinding stones was reported [38]. Burning, chewing, heating/roasting, pounding, and boiling or soaking in hot or cold water and milk were reported, and these are then orally administered as is the case with Western medicine [38]. Preparations for application onto the skin such as ointments, poultices, and liniments are frequently percutaneous, by rubbing or covering which are occasionally complimented by massage [38]. Rarely are antimalarial remedies administered through the nasal route. Fresh solid materials are eaten and chewed directly upon collection or after initial pounding/crushing. Dry plant materials are smoked and inhaled. These findings corroborate observations in other countries [33, 90–92].

Malaria is caused by protozoan intracellular haemoparasites, and its treatment entails delivering adequate circulating concentration of appropriate antiprotozoal chemicals. The oral route is a convenient and noninvasive method of systemic treatment as it permits relatively rapid absorption and distribution of active compounds from herbal remedies, enabling the delivery of adequate curative power [93]. In addition, potential risk of enzymatic breakdown and microbial fermentation of active chemical entities may prompt the use of alternative routes of herbal remedy administration like inhalation of the steam or rubbing on the skin.

In this survey, it was noted that few plant species are used for management of malaria simultaneously in different locations. This could probably be attributed to the abundant distribution of the analogue active substances among species, especially belonging to family Asteraceae, Euphorbiaceae, Fabaceae, Meliaceae, Rubiaceae, and Rutaceae. Differences in geographical and climatic conditions may also influence the flora available in a given region. However, some plants have a wider distribution and therefore are used by most communities [34].

### 3.3. Perception, Prevention, and Treatment of Malaria and Its Symptoms

In rural Kenya, some believe that *esse* (malaria in native Tugen dialect) is caused by *Cheko che makiyo* (fresh unboiled milk), dirty water, *ikwek* (vegetables such as *Solanum nigrum* and *Gynadropis gynadra*) [54], mosquito bites, or cold weather [42]. Thus, burning of logs and plants such as *Albizia coriaria* with cow dung, *Azadirachta indica* (L) Burm (fresh leaves), *Ocimum basilicum* L., *Ocimum suave* Willd. (fresh leaves), and *Plectranthus barbatus* Andr. (ripe fruits or seeds) are done to keep mosquitoes away [17, 42]. *Artemisia annua* L. is planted in the home vicinity or near the bedroom window to repel mosquitoes believed to cause malaria [42].

Except in the case of life-threatening illnesses or where there is concern that there may be some supernatural forces in the aetiology of the disease, malaria and its symptoms (periodic fever, sweating, headache, backache, and chills) are treated primarily using decoctions and infusions of plants. Whenever it is thought that malaria is due to supernatural forces, diviners (such as *Orgoiyon* among the Tugen and *Oloiboni* among the Maasai) are consulted [94]. *Croton dichogamus* Pax though used for normal malaria treatment is used by *Oloiboni* for treatment of malaria or other ailment(s) thought to be due to witchcraft [22]. According to indigenous diagnoses, malaria is due to the presence of excess bile in the body, so the bile has to be expelled before healing can take place. Thus, purgation is regarded as the key treatment regimen for malaria [22, 54].

On the basis of this knowledge, different forms of herbal medications are prescribed according to the severity of the illness. Treatment of malaria is based on a number of interlinked elements: beliefs related to causation, the action or effectiveness of “modern” medicines, and the availability of plant treatments [54]. *Salvadora persica* L. is used for management of malarial colds, while *Aneilema spekei* (C. B. Clarke) is used for prevention of malaria fever [22]. The whole plant is mixed with other herbs in milk and sprinkled onto the patient. This is often administered by an *Oloibon* among the Maasai [22].

Though single plant parts are often used, more than one plant part, for example, decoctions from a mixture of roots of *Plectranthus sylvestris* together with those of *Cassia didymobotrya* and *Clerodendrum johnstonii* may be used as a remedy for malaria and headache [52]. *Acacia* species stem bark was reported to be used as a first treatment and is usually prepared as an overnight cold-water infusion, and then 40 ml is taken three times a day [11]. A follow-up medication would involve taking a decoction made from powders of *Aloe* species (leaf juice), *Rhamnus staddo* (stem or root bark), *Clerodendrum myricoides* (root bark), *Warburgia ugandensis*, *Teclea nobilis* (stem barks), and *Caesalpinia volkensii*, *Ajuga remota* Benth, *Rhamnus prinoides,* and *Azadirachta indica* leaves [11]. For this, 40 ml is taken thrice a day for 5 days.

The popular method of preparation as decoctions and concoctions suggest that the herbal preparations may only be active in combination, due to synergistic effects of several compounds that are inactive singly [95]. It is possible that some of the compounds that are inactive *in vitro* could exhibit activity *in vivo* due to enzymatic transformation into potent prodrugs [96] as reported for *Azadirachta indica* extracts [97].

### 3.4. Adverse Side Effects, Antidotes, and Contraindications of Medicinal Plants in Kenya

In traditional context, the pharmacological effect of medicinal plants is generally ascribed to their active and “safe” content that will only exert quick effect when taken in large quantities [22, 33]. Most reviewed reports in this study did not mention the side effects of antimalarial preparations. Nevertheless, herbal preparations from some antimalarial plants were reported to induce vomiting, diarrhea, headache, and urination [22, 54] (Table 2). This may be due to improper dosage, toxic phytochemicals, or metabolic by-products of these preparations [22].

However, purgation and emesis are interpreted as signs that malaria is leaving the body and that the healing process has begun [22, 54]. It is reasonable that some side effects might also be masked through the use of more than one plant (or plant parts) especially for bitter remedies (such as *Ajuga remota* Benth.) [11, 38]. However, some herbalists are known to use more than one plant (plant parts) as a trick of keeping the secrecy of their formula [11]. Boiling of plant parts in goat fat, meat bone broth (as is done for *Carissa edulis*), taking decoctions mixed with milk (for *Rhamnus prinoides*), and mixing remedies with milk and salt for *Salvadora persica* L. [22] could serve as antidotes for potential side effects from use of the herbal preparations as reported elsewhere [33]. Some of the plants reported in this study such as *Ajuga integrifolia* and *Croton macrostachyus* were reported in Ethiopia to cause vomiting, nausea, headache, urination, and diarrhea when used for management of malaria [33]. Because the outcome of the treatment remains generally unclear due to lack of feedback from patients, herbalists rely on anecdotal reporting as far as efficacy and side effects are concerned.

Some antimalarial plants were reported as contraindicated to pregnant women and children (Table 2). Gathirwa et al. [50] reported that the posology of antimalarial herbal preparations in Kenya sometimes is dictated by the plant to be used, the traditional herbalist, the sex and the age of the patient, reiterating that pregnant women and children are often given lower dosages compared to other adults. This indicates the existence of research gaps with regard to the potential toxicities and corresponding counteracting mechanisms of antimalarial plants in Kenya. This gap represents a barrier to effective development and exploitation of indigenous antimalarial plants. In essence, some of the plants listed are reported to exhibit marked toxicity. *Teclea simplicifoli* (roots) is regarded to be poisonous by rural Kenyans [98]. *Catharanthus roseus* (L.) G. Don is another such plant known to house neurotoxic alkaloids [99]. Vincristine and vinblastine in this plant are highly cytotoxic antimitotics that block mitosis in metaphase after binding to mitotic microtubules [100]. Side effects such as kidney impairment, nausea, myelosuppression, constipation, paralytic ileus, ulcerations of the mouth, hepatocellular damage, abdominal cramps, pulmonary fibrosis, urinary retention, amenorrhoea, azoospermia, orthostatic hypotension, and hypertension [101–103] have been documented for antitumor drugs vincristine and vinblastine derived from this plant. These observations could partly explain why some antimalarial herbal preparations in Kenya are ingested in small amounts, applied topically, or are used for bathing. This gives a justification for the investigation of the plants for their potential toxicity.

### 3.5. Other Ethnomedicinal Uses of Antimalarial Plants Used in Rural Kenya

Most of the antimalarial plant species identified are used for traditional management of other ailments in Kenya and in other countries. *Ajuga remota* Benth (different parts), for example, are used to relieve toothache, severe stomachache, oedema associated with protein-calorie malnutrition disorders in infants when breast-feeding is terminated, pneumonia, and liver problems [52, 104, 105]. Such plants are used across different ethnic communities for managing malaria and can be a justification of their efficacy in malaria treatment [19].

### 3.6. Toxicity, Antiplasmodial, and Antimalarial Studies

Table 3 shows the list of some of the antimalarial plants used in Kenya with reports of toxicity/safety, antimalarial, and antiplasmodial activity evaluation. Across African countries, many antimalarial plants captured in this review have demonstrated promising therapeutic potential on preclinical and clinical investigations [68, 106–111]. Interestingly, antimalarial compounds have been identified and isolated from some of these species [62, 112].

Export of indigenous medicinal plants bring substantial foreign exchange to African countries such as Egypt [113], South Africa [114], Uganda, Tanzania, and Kenya [115]. Despite the success of traditional practices and abundance of indigenous medicinal plants (Table 1), antimalarial plants research in Kenya stops mostly on ethnobotanical surveys, with extensions limited to evaluation of crude extracts from plants against *Plasmodium berghei* [48, 56, 71]. A gap is evident with regard to research geared towards identifying and isolating plant bioactive compounds and establishing the efficacy and safety of medicinal plants through *in vitro* assays using human *Plasmodium* parasites and *in vivo* assay involving higher animal models and randomized clinical trials [50]. For example, the toxicity of 16,17-dihydrobrachycalyxolid isolated in *Vernonia brachycalyx* has been reported to be due to its ability to inhibit the proliferation of phytohaemmaglutinin-treated human lymphocytes [116]. A median inhibitory concentration (IC_50_) of 7.8 *μ*g/ml was reported, which is comparable to the median concentration obtained in the antiplasmodial assay by Oketch-Rabah et al. [58] (Table 3). To assess whether observed antiplasmodial activities are due to a specific or a general toxicity effect, the experimental selectivity index (SI) needs to be calculated for extracts and only a few studies in Kenya has attempted this [48–50]. It is worth noting that there is always a variation in the degree of toxicity depending on the sensitivity of the animals, tissue, or cells used, type of extract, nature of the test substance, dose, and mode of administration. In this study, 38.8% (54/139) of the total plants were evaluated for their toxicities. Of these, 41 showed low cytotoxicity with LC_50_ > 20 *μ*g/ml. Some of these plants such as *Artemisia annua*, *Carica papaya*, *Flueggea virosa,* and *Schkuhria pinnata* fortuitously showed good antimalarial activity. On the contrary, extracts of some plants used for malaria treatment with good activity are potentially toxic, for example, dichloromethane leaf extract of *Microglossa pyrifolia,* methanolic extract of *Uvaria acuminata* (CC_50_ = 2.37 *μ*g/ml), and petroleum ether leaf extract of *Vernonia amygdalina.*

In total, 139 (48.6%) of the species identified have been investigated for antiplasmodial (*n* = 25, 18%) or antimalarial activities (*n* = 135, 97.1%). However, there is no record on antiplasmodial or antimalarial activity of about 51.4% of the species used although they could be potential sources of antimalarial remedies. In the antiplasmodial activity, parasite suppression ranged from 3.5 to 5.2% in *Leucas calostachys* Olive aqueous leaf extracts [82] to 90% in *Ajuga integrifolia* aqueous leaf extracts [177]. In antimalarial studies against chloroquine-sensitive (D6, 3D7, D10, FCA/GHA, FCR3, K39, and NF54) and chloroquine-resistant (DD2, ENT 30, FCR3, K1, V1/S, and W2) *P. falciparum* isolates, 49.6% (67/135) were active with the lowest IC_50_ of 0.16 *μ*g/ml recorded against NF54 isolate for spermine alkaloids in *Albizia gummifera* [178]. On the other hand, 68 species (50.4%) were inactive. The most active extracts were those of isolated pure compounds. For example, spermine alkaloids: budmunchiamine K, 6-hydroxybudmunchiamine K, 5-normethylbudmunchiamine K, 6-hydroxy-5-normethylbudmunchiamine K, and 9-normethylbudmunchiamine K from *Albizia gummifera* bark [178] had IC_50_ of 0.16 *μ*g/ml recorded against ENT30. Curine, isolated from *Cissampelos mucronate* roots, showed antimalarial activity against W2 isolate with IC_50_ of 0.24 *μ*g/ml [74]. At present, *Artemisia annua* [106, 107], *Azadirachta indica* [108], and *Vernonia amygdalina* [111] have been subjected to clinical studies. Artemisinin from *Artemisia annua* is an ingredient of artemisinin-based combination therapy currently recommended for treatment of malaria [124]. As identified earlier, few clinical trials have been done on antimalarial plants. This is partly due to the regulatory requirements for clinical studies, as well as the financial input required.

## 4. Conclusion

Indigenous knowledge on medicinal plants in Kenya is a good resource for malaria management. However, further studies are required to isolate the active compounds in the unstudied plants which can be used to standardize plant materials so as to install a reproducible herbal medicine practice. Safety and toxicity as well as clinical studies are required as some of the plants are used as admixtures in traditional herbal management of malaria.

## Figures and Tables

**Table 1:** Synopsis of medicinal plants used in the management of malaria in Kenya.

Plant family	Botanical name	Local name	Part(s) used	Habit	Preparation mode	Reference(s)
Acanthaceae	*Justicia betonica* L.	Shikuduli	Aerial parts	Herb	Decoction	[34, 35]

Alliaceae	*Allium sativum* L.	Kitungu saumu (Luo)	Roots	Herb	Crushed, chewed	[36]

Aloeaceae	*Aloe barbadensis* Mill. (vera)	Oldopai (Maasai)	Leaves	Herb	Not specified	[37]
	*Aloe kedongensis* Reynolds	Osukuroi (Maasai)	Leaves, roots	Herb	Infusion	[3, 38–40]
	*Aloe elgonica* Bullock	Not reported	Leaves, roots	Herb	Decoction	[41]
	*Aloe lateritia* Engl.	Kiiruma (Kikuyu)	Leaves, root	Herb	Decoction	[3, 42]
	*Aloe volkensii* Engl.	Osukuroi (Maasai)	Leaves	Herb	Decoction	[22]
	*Caesalpinia volkensii* Harms	Mujuthi (Kikuyu)	Leaves	Liana	Decoction	[3, 11, 43, 44]

Amaranthaceae	*Achyranthes aspera* L.	Uthekethe (Kamba)	Whole plant	Herb	Not specified	[23, 45]
	*Amaranthus hybridus* L.	Mchicha (Swahili)	Leaves	Herb	Decoction	[17, 46]
	*Celosia schweinfurthiana* Schinz.	Not reported	Not specified	Shrub	Not specified	[47]
	*Cyathula schimperiana* non Moq	Namgwet	Leaves, roots	Herb	Decoction	[38, 40]
	*Cyathula cylindrica* Moq	Ng'atumyat	Roots	Herb	Decoction	[38, 40]
	*Sericocomopsis hildebrandtii* Schinz.	Oloituruj-ilpeles (Maasai)	Roots	Shrub	Decoction	[22, 48]

Anacardiaceae	*Heeria insignis* Del.	Mwamadzi (Swahili)	Bark, stem bark	Tree	Decoction	[17, 46]
	*Lannea schweinfurthii* (Engl.) Engl.	Mnyumbu	Bark, leaves	Shrub	Not specified	[49, 50]
	*Ozoroa insignis* Delile	Not reported	Not reported	Shrub	Not specified	[42]
	*Rhus natalensis* Bernh. ex Krauss	Muthigiu (Kikuyu)	Root, stem, fruits, root bark	Tree	Decoction	[3, 42, 49–51]
	*Rhus vulgaris* Meikle	Sungula	Leaves	Herb	Decoction	[3, 42]
	*Sclerocarya birrea* (A. Rixh.) Hochst	Oloisuki (Maasai)	Bark	Tree	Not specified	[49]
	*Searsia natalensis* (Bernh. ex C. Krauss)	Olmisigiyioi (Maasai)	Leaves	Herb	Decoction	[34]

Annonaceae	*Uvaria acuminata* Oliv.	Mukukuma (Kamba)	Roots	Shrub	Not specified	[50]
	*Uvaria scheffleri* Diels	Not reported	Leaves	Liana	Decoction	[17]
Apiaceae	*Centella asiatica* (L.) Urb.	Not reported	Leaves	Herb	Decoction	[17]

Apocynaceae	*Carissa edulis* (Forssk.) Vahl.	Olamuriaki (Maasai), Mukawa (Kikuyu)	Root, root bark	Shrub	Decoction, inhale steam	[3, 17, 34, 38, 40, 47, 48, 52, 53]
	*Catharanthus roseus* (L.) G. Don	Olubinu	Not specified	Herb	Not specified	[47]
	*Gomphocarpus fruticosus* (L.) W. T. Aiton	Kosirich	Root	Herb	Not specified	[54]
	*Laudolphia buchananii* (Hall.f) Stapf	Mhonga (Swahili)	Leaves	Liana	Decoction	[17, 46]
	*Mondia whitei*	Ogombo (Luo)	Roots	Herb	Chewed	[42]
	*Rauwolfia cothen*	Not reported	Root bark	Shrub	Decoction	[17]
	*Saba comorensis* (Bojer ex A.D.C) Pichon	Abuno (Luo)	Not specified	Herb	Not reported	[42]

Asclepiadaceae	*Curroria volubilis* (Schltr.) Bullock	Simatwet	Bark	Liana	Decoction	[38, 40]
	*Periploca linearifolia* Dill. & A. Rich.	Muimbathunu (Kikuyu)	Bark	Liana	Decoction	[3, 44]

Asteraceae	*Achyrothalamus marginatus* O. Hoffm.	Not reported	Leaves	Herb	Decoction	[55]
	*Acmella caulirhiza* Del.	Shituti	Aerial parts	Shrub	Decoction	[34, 56]
	*Ageratum conyzoides* L.	Not reported	Whole plant	Herb	Decoction	[56, 57]
	*Artemisia afra* Jacq	Not reported	Leaves	Shrub	Decoction	[41]
	*Artemisia annua* L.	Not reported	Leaves	Shrub	Decoction	[42]
	*Aspilia pluriseta* Schweinf.	Rirangera	Leaves	Herb	Decoction	[35]
	*Bidens pilosa* L.	Nyanyiek mon (Luo)	Leaves	Herb	Decoction	[11, 37]
	*Ethulia scheffleri* S. Moore	Not reported	Leaves	Herb	Decoction	[58]
	*Gutenbergia cordifolia* Benth.	Olmiakaru-kewon (Maasai)	Leaves	Herb	Decoction	[48]
	*Kleinia squarrosa*	Mungendya (Kamba)	Leaves	Shrub	Infusion	[55]
	*Launaea cornuta* (Oliv and Hiern) C. Jeffrey	Uthunga (Kamba)	Leaves	Liana	Infusion/decoction	[17, 46, 55]
	*Microglossa pyrifolia* (Lam.) O. Kuntze	Nyabung-Odide (Luo)	Root, leaves	Shrub	Decoction	[34, 37, 38]
	*Psiadia arabica* Jaub. & Pach	Nyabende winy (Luo)	Not specified	Herb	Not specified	[42]
	*Psiadia punctulata* (D.C.) Vatke	Olobai (Maasai)	Roots	Herb	Not specified	[48]
	*Sonchus schweinfurthii* Oliv. & Hiern	Egesemi (Kuria)	Not specified	Herb	Not specified	[37]
	*Schkuhria pinnata* (Lam.) Kuntze ex Thell	Gakuinini (Kikuyu)	Whole plant	Herb	Infusion	[3, 23, 42, 44]
	*Senecio syringitolia* O. Hoffman	Reisa (Digo)	Leaves	Herb	Decoction	[17, 46]
	*Solanecio mannii* (Hook. f) C. Jeffrey	Maroo, marowo (Luo), Livokho	Leaves	Shrub	Decoction	[23]
	*Sonchus luxurians* (R.E. Fries) C. Jeffrey	Kimogit (Nandi)	Roots	Herb	Decoction	[38]
	*Sphaeranthus suaveolens* (Forsk.) DC	Njogu-ya-iria	Whole plant	Herb	Infusion, rubbed on the body	[44, 52]
	*Tithonia diversifolia* (Hemsl.) Gray	Maua madongo (Luo)	Leaves	Shrub	Decoction	[3, 34, 42]
	*Tridax procumbens L.*	Not reported	Whole plant	Herb	Infusion	[17]
	*Vernonia amygdalina* Del.	Musulilitsa	Leaves	Shrub	Decoction	[17, 34, 42]
	*Vernonia auriculifera* (Welw.) Hiern	Muthakwa	Leaves, roots, bark	Shrub	Infusion, decoction	[35, 37, 38, 41, 44]
	*Vernonia brachycalyx* O. Hoffm. Schreber	Irisabakw (Kuria)	Leaves	Herb	Decoction	[37, 44, 58]
	*Vernonia brachycalyx O.* Hoffm. Lasiopa Lam.	Olusia (Luo)	Leaves	Herb	Decoction	[37]
	*Vernonia lasiopus* O. Hoffm.	Shiroho, Mwatha	Leaves, root bark	Shrub	Infusion	[23, 35, 44]

Bignoniaceae	*Kigelia africana* (Lamk.) Benth.	Omurabe, Morabe	Leaves, bark, fruits	Tree	Decoction	[44, 58, 59]
	*Markhamia lutea* (Benth.) K. Schum.	Lusiola, Shisimbali	Bark	Tree	Decoction	[34, 47]
	*Markhamia platycalyx* Sprague	Siala (Luo)	Not specified	Tree	Not specified	[42]
	*Spathodea campanulata* P. Beauv.	Muthulio, Mutsuria	Leaves	Tree	Decoction	[34]

Boraginaceae	*Ehretia cymosa* Thonn	Mororwet	Leaves, roots	Shrub	Infusion	[38, 40]

Burseraceae	*Commiphora eminii* Engl	Mukungugu (Kikuyu)	Not specified	Tree	Not specified	[3]
	*Commiphora schimperi* (Berg) Engl.	Osilalei (Maasai), Dzongodzongo (Swahili)	Inner bark, roots, stem bark	Tree	Decoction	[17, 46, 48]

Canellaceae	*Warburgia salutaris* (Bertol.F.) Chiov.	Osokonoi (Maasai)	Bark	Tree	Decoction	[22, 37, 45]
	*Warburgia stuhlmannii Engl.*	Not reported	Stem bark	Tree	Decoction	[17]
	*Warburgia ugandensis* Sprague subsp ugandensis	Muthiga (Kikuyu)	Stem bark, fruits, leaves	Tree	Decoction	[3, 11, 22, 34, 43, 51, 54]

Capparaceae	*Boscia angustifolia* A. Rich.	Oloiroroi (Maasai)	Inner bark fibres, stem bark	Tree	Decoction	[42, 44, 48, 52]
	*Boscia salicifolia* Oliv.	Mwenzenze (Kamba)	Not specified	Tree	Not specified	[49]
	*Cadaba farinosa* Forssk	Akado marateng (Luo)	Not specified	Shrub	Not specified	[42]
Capparidaceae	*Cleome gynandra* L.	Isakiat	Leaves, roots	Herb	Decoction	[40]
Cariaceae	*Carica papaya* L.	Poipoi, Apoi (Luo)	Leaves, roots, sap	Shrub	Infusion, decoction	[36]

Celastraceae	*Maytenus arbutifolia* (A. Rich.) Wilczek	Muraga	Root bark	Shrub	Decoction	[44]
	*Maytenus heterophylla* (Eckl. & Zeyh.) N. Robson	Muraga	Root, root bark	Shrub	Decoction	[41, 44]
	*Maytenus putterlickioides* (Loes.) Excell & Mendonca	Muthuthi	Root bark	Shrub	Decoction	[44]
	*Maytenus senegalensis* (Lam.) Exell	Muthuthi (Kikuyu)	Not specified	Shrub	Not specified	[3, 47]
	*Maytenus undata* (Thunb.) Blakelock	Muthithioi	Root bark, leaves	Shrub	Decoction	[44]

Cleomaceae	*Cleome gynandra* L.	Isakiat	Leaves roots	Herb	Decoction	[38]

Combretaceae	*Combretum illairii* Engl.	Mshinda arume	Leaves, root bark	Tree	Decoction	[50]
	*Combretum molle* G. Don	Muama, Kiama (Kamba)	Bark, leaves	Tree	Decoction	[17, 45]
	*Combretum padoides* Engl. & Diels	Mshinda arume	Leaves, roots	Tree	Decoction	[17, 46, 50, 60]
	*Terminalia brownii* Fresen.	Muuku (Kamba)	Bark	Tree	Decoction	[55]
	*Terminalia spinosa* Engl.	Not reported	Bark, stem bark	Tree	Decoction, infusion	[17, 61]

Commelinaceae	*Aneilema spekei* (C. B. Clarke)	Enkaiteteyiai (Maasai)	Whole plant	Liana	Decoction	[22]
	*Commelina forskaolii* Vah	Not reported	Not specified	Herb	Not specified	[47]

Crassulaceae	*Kalanchoe lanceolata* (Forsk.) Pers.	Mahuithia (Kikuyu)	Not specified	Herb	Not specified	[3]

Cucurbitaceae	*Cucumis aculeatus* Cogn.	Gakungui (Kikuyu)	Leaves	Climber	Decoction	[3, 34, 42, 62]
	*Cucumis prophetarum* L.	Chepsawoy (Kipsigis)	Root tuber	Herb	Decoction	[39]
	*Gerranthus lobatus* (Cogn.) Jeffrey	Mgore manga (Digo)	Leaves, roots	Herb	Decoction	[17, 46]
	*Momordica foetida* Schumach	Cheptenderet (Kipsigis)	Leaves, roots	Liana	Decoction, roasting	[17, 38, 41]
	*Momordica friesiorum* Hams C. Jeffrey	Libobola	Root tuber	Herb	Decoction	[54]
	*Zehneria minutiflora* (Cogn.) C. Jeffrey	Manereriat (Kimanererit)	Leaves, roots	Liana	Decoction	[38]

Cyperaceae	*Cyperus articulatus* L.	Ndago	Tuber	Herb	Infusion	[44]

Ebenaceae	*Euclea divinorum* Hiern	Uswet (Markweta)	Root bark	Tree	Decoction, use for brushing teeth	[38, 47]
	*Diospyros abyssinica* (Hiern) F. White subsp. abyssinica	Lusui	Bark	Tree	Decoction	[41, 59]
	*Diospyros scabra*	Not reported	Bark	Tree	Decoction	[61]

Euphorbiaceae	*Bridelia micrantha* Baill. (Hochst).	Mdungu (Digo)	Leaves, bark, stem bark	Shrub	Decoction	[17, 46]
	*Clutia abyssinica* Jaub. & Spach	Muthima mburi (Kikuyu)	Leaves, root, root bark	Shrub	Decoction	[3, 38, 44]
	*Croton dichogamus* Pax.	Oloiborrbenek (Maasai)	Whole plant	Shrub	Decoction	[22, 38]
	*Croton macrostachyus* Hochst. ex Del.	Mukinduri (Kikuyu)	Leaves, root, bark	Tree	Decoction	[34, 38, 56]
	*Croton megalocarpoides* Friis & M.G. Gilbert	Ormegweit (Maasai)	Bark	Tree	Decoction	[22]
	*Croton megalocarpus* Del.	Not reported	Not specified	Tree	Not specified	[3]
	*Euphorbia inaequilatera* Sond.	Ogota Kwembeba	Whole plant	Shrub	Decoction	[35]
	*Euphorbia meridionalis* Bally & S. Carter	Enkokuruoi (Maasai)	Stem	Climber	Not specified	[22]
	*Euphorbia tirucalli* L.	Kariria (Kikuyu)	Not specified	Tree	Not specified	[3]
	*Flueggea virosa* (Willd.) Voigt	Mukwamba	Root bark	Tree	Decoction	[50]
	*Flueggea virosa* (Roxb.ex Willd.) Royle	Mkwamba, mteja (Swahili)	Aerial parts, root bark	Shrub	Decoction	[17, 34]
	*Neoboutonia macrocalyx* Pax	Mutuntuki	Leaves, stem bark	Tree	Decoction	[44, 53]
	*Phyllanthus sepialis* Müll. Arg.	Not reported	Leaves	Shrub	Decoction	[34]
	*Ricinus communis* L.	Kivaiki (Kamba)	Root, seeds, leaves	Shrub	Decoction, topical	[17, 38, 46]
	*Sapium ellipticum*	Achak (Luo)	Not specified	Shrub	Not specified	[42]
	*Suregada zanzibariensis* Baill	Not reported	Root bark	Shrub	Decoction	[17]

Fabaceae	*Abrus precatorius* L. ssp africanus Verdc	Ndirakalu	Leaves	Herb	Not specified	[42, 50]
	*Acacia hockii* De Wild.	Eluai (Maasai)	Root bark	Tree	Decoction	[48]
	*Acacia mellifera* (M.Vahl) Benth.	Oiti (Maasai), Muthiia (Kamba)	Stem bark, root, pith	Tree	Decoction	[11, 22, 48, 52, 63]
	*Acacia nilotica* (L.) Willd.ex Delile	Olkirorit, Ol-rai (Masaai)	Bark, root	Tree	Decoction	[22, 37, 53, 64]
	*Acacia oerfota* (Forssk.) Schweinf.	Not reported	Root	Tree	Not reported	[63]
	*Acacia seyal* Delile	Mgunga (Digo)	Root	Tree	Decoction	[17]
	*Acacia tortilis* (Forssk.) Hayne	Oltepesi (Maasai)	Sap, roots	Tree	Taken directly, decoction	[22, 48]
	*Albizia amara* (Roxb.) Boiv.	Mwiradathi	Stem bark	Tree	Decoction	[44]
	*Albizia anthelmintica* Brongn.	Kyoa (Kamba)	Root, bark	Tree	Decoction	[17, 22, 63]
	*Albizia coriaria* Welw ex Oliver	Omubeli	Multiple parts	Tree	Decoction	[42, 47, 57, 65]
	*Albizia gummifera* (J.F. Gmel.)	Seet (Nandi)	Root, stem bark	Tree	Decoction	[23, 34, 38, 42, 44, 66]
	*Albizia zygia* (DC) J.F. Macbr.	Ekegonchori (Kuria)	Not specified	Tree	Not specified	[37]
	*Cassia didymobotrya* Fres.	Irebeni (Kuria), Murao	Leaves, roots, root bark	Shrub	Infusion, decoction	[37, 38, 40, 44]
	*Cassia occidentalis* L.	Mnuka uvundo (Swahili)	Leaves, roots	Herb	Decoction	[11, 17, 46]
	*Dichrostachys cinereal* L.	Chinjiri (Digo)	roots	Tree	Decoction	[17]
	*Erythrina abyssinica* Lam. ex DC.	Omutembe (Kuria), Muhuti (Kikuyu)	Root, bark	Tree	Decoction	[3, 23, 34, 37, 38, 42]
	*Indigofera arrecta* A. Rich	Not reported	Roots	Herb	Decoction, chew directly	[41]
	*Mucuna gigantea*	Ogombo (Luo)	Not specified	Liana	Not specified	[42]
	*Senna didymobotrya* (Fresen) Irwin & Barneby	Osenetoi (Maasai)	Roots, leaves, bark, stem	Shrub	Decoction	[3, 23, 34, 41, 42, 67]
	*Senna occidentalis* (L.) Link	Imbindi	Roots	Shrub	Decoction	[34, 47]
	*Tamarindus indica* L.	Muthumula (Kamba), Mkwadzu (Swahili)	Bark, fruits, roots, leaves	Tree	Decoction, fruit eaten	[17, 46, 47, 54]
	*Tylosema fassoglense*	Not reported	Tuber	Climber	Not specified	[56]

Hydnoraceae	*Hydnora abyssinica* Schweinf.	Muthigira (Kikuyu)	Not specified	Herb	Not specified	[3]

Hypericaceae	*Harungana madagascariensis* Lam. ex Poir.	Musila	Stem bark	Tree	Decoction	[17, 34, 42]

Icacinaceae	*Pyrenacantha malvifolia* Engl.	Empalua (Maasai)	Roots	Climber	Not specified	[22]

Lamiaceae	*Ajuga integrifolia* Buch. Ham.	Imbuli yumtakha	Aerial parts	Herb	Decoction	[34]
	*Ajuga remota* Benth.	Wanjiru (Kikuyu)	Leaves, roots, whole plant	Herb	Decoction	[3, 11, 23, 38, 44, 68, 69]
	*Clerodendrum johnstonii* Oliv	Singoruet (Nandi)	Leaves	Shrub	Infusion	[34, 38]
	*Fuerstia africana* T.C.E.Fr.	Kwa matsai, aremo (Luo)	Aerial parts, leaves, whole plant	Herb	Decoction, infusion	[34, 38, 44, 48, 65]
	*Hoslundia opposita* Vahl.	Cheroronit, Cherungut (Nandi)	Leaves, whole plant	Shrub	Decoction	[17, 38, 46, 50]
	*Leucas calostachys* Oliv	Bware (Luo), Lumetsani	Leaves, roots, aerial parts	Shrub	Decoction	[34, 37, 38]
	*Leucas martinicensis* (Jacq.) Ait.f.	Chepkari (Nandi)	Flowers	Herb	Infusion	[38]
	*Leonotis mollissima* Guerke	Nyanyondhi (Luo), Orbibi (Maasai)	Leaves, roots	Shrub	Decoction	[23, 37, 38]
	*Leonotis nepetifolia* (R. Br) Ait.f.	Kipchuchuniet (Kipsigis)	Not specified	Shrub	Decoction	[47, 70]
	*Ocimum basilicum* L.	Sisiyat (Nandi)	Leaves	Herb	Decoction	[23, 46]
	*Ocimum balansae* Briq.	Not reported	Leaves	Herb	Decoction	[17]
	*Ocimum gratissimum* L. Suave wiild, *O. tomentosum* Oliv.	Mukandu (Kamba)	Leaves	Herb	Decoction	[17, 23]
	*Ocimum kilimandscharicum* Guerke	Mutaa (Kamba)	Aerial parts	Herb	Inhale steam	[3, 34, 56]
	*Ocimum lamiifolium* Benth	Not reported	Roots	Shrub	Decoction	[38]
	*Ocimum suave* Willd	Murihani (Giriama)	Leaves	Herb	Decoction	[17, 46, 71]
	*Plectranthus barbatus* Andrews	Kan'gurwet (Markweta)	Leaves	Shrub	Infusion, decoction	[17, 34, 42, 46, 56, 58]
	*Plectranthus sylvestris* Gurke	Not reported	Leaves	Herb	Not specified	[58]
	*Rotheca myricoides* (Hochst.) Steane and Mabb (*Clerodendrum myricoides* (Hochst.) Vatke)	Olmakutukut (Maasai), Munjuga iria (Kikuyu)	Roots, leaves, root bark	Shrub	Decoction	[17, 34, 38, 42, 44, 48, 67]

Lauraceae	*Ocotea usambarensis* Engl.	Muthaiti (Kikuyu)	Root bark	Tree	Infusion	[3, 44]

Loganiaceae	*Strychnos henningsii* Gilg	Muteta (Kamba, Kikuyu)	Roots, leaves, stem bark	Tree	Decoction	[3, 11, 44, 47, 55, 67]

Malvaceae	*Adansonia digitata* L.	Mbamburi (Swahili)	Leaves	Tree	Decoction	[17, 46]
	*Azanza gackeana* (F. Hoffm.) Excell & Hillcoat	Mutoo (Kikuyu)	Not specified	Tree	Not specified	[3]
	*Grewia bicolor* Juss	Esiteti (Maasai)	Not specified	Shrub	Not specified	[47]
	*Grewia hainesiana* Hole	Not reported	Leaves	Shrub	Decoction	[17]
	*Grewia hexamita* Burret	Mkone (Digo)	Roots, leaves	Shrub	Decoction	[46]
	*Grewia plagiophylla* K. Schum	Mkone (Digo)	Bark, leaves		Not specified	[50]
	*Grewia trichocarpa* (Hochst) ex A. Rich.	Cone (Digo)	Roots	Shrub	Decoction	[17, 41, 46]
	*Pavonia kilimandscharica* Gurke	Chemanjililiet, Chepsabuni (Nandi)	Roots	Herb	Decoction	[38]
	*Sida cordifolia* L.	Menjeiwet (Nandi)	Leaves	Shrub	Infusion	[38]
Meliaceae	*Azadirachta indica* A. Juss	Muarubaini (Kamba)	Leaves, roots, bark	Tree	Decoction, inhalation, topical	[3, 11, 17, 36, 43, 50, 54, 55, 72]
	*Azadirachta indica* (L) Burm.	Mkilifi (Digo)	Leaves, roots, root bark	Tree	Decoction	[46, 73]
	*Ekebergia capensis* Sparrm.	Olperre-longo (Maasai)	Bark	Tree	Decoction	[3, 48]
	*Melia azedarach* L.	Mwarubaine	Not specified	Tree	Not specified	[47]
	*Melia volkensii* L.	Mukau (Kamba)	Bark	Tree	Decoction	[55]
	*Melia azedarach* L.	Mwarubaini (Nandi)	Leaves, bark	Tree	Decoction	[34, 38, 42]
	*Trichilia emetica* Vahl.	Munyama	Bark	Tree	Decoction	[34, 72]
	*Turraea mombassana* C. DC	Onchani Orok (Maasai)	Leaves, root, fruits	Shrub	Decoction	[67]
	*Turraea robusta*	Not reported	Root bark	Shrub	Decoction	[49]

Melianthaceae	*Bersama abyssinica* Fres.	Kibuimetiet (Nandi)	Root bark, bark, seeds	Tree	Decoction	[38, 41]

Menispermaceae	*Cissampelos mucronata* A. Rich.	Mukoye	Root	Climber	Root chewed	[17, 34, 74, 75]
	*Cissampelos pareira* L.	Karigi munana	Root, root bark	Liana	Decoction	[39]

Moraceae	*Ficus bussei* Warb ex Mildbr and Burret	Mgandi (Digo)	Roots, leaves	Tree	Decoction	[17, 46]
	*Ficus cordata* Thunb	Oladardar (Maasai)	Branches, roots, stem	Tree	Decoction	[67]
	*Ficus sur.* Forssk	Omora	Stem bark	Tree	Decoction	[35]
	*Ficus thonningii* Blume	Mutoto	Stem bark	Tree	Decoction	[34]

Myricaceae	*Myrica salicifolia* A. Rich.	Murima	Root bark	Tree	Decoction	[44]

Myrsinaceae	*Embelia schimperi* Vatke	Kibong'ong'inik (Nandi)	Seeds	Tree	Decoction	[38]
	*Maesa lanceolata* Forssk	Katera (Luo), Kibabustanyiet (Nandi)	Roots, fruits, seeds, bark	Shrub	Decoction	[22, 34, 38, 76]

Myrtaceae	*Eucalyptus globulus* Labil.	Mubau (Kikuyu)	Not specified	Tree	Not specified	[3]
	*Psidium guajava* L.	Mapera (Luo)	Leaves, fruits	Tree	Decoction	[36]

Oleaceae	*Jasminum floribunda* R.Br.	Not reported	Root	Herb	Decoction	[41]
	*Jasminum fluminense* Vell.	Kipkoburo	Bark, stem, root tuber	Vine	Not specified	[77]
	*Olea capensis* L.	Mutukhuyu, Mucharage	Stem bark	Tree	Decoction	[41, 44]
	*Olea europaea* L.	Oloirien (Maasai)	Inner/stem bark	Tree	Decoction	[3, 22, 44, 45, 48]
	*Ximenia americana* L.	Olamai (Maasai)	Leaves	Tree	Decoction	[47]

Onagraceae	*Ludwigia erecta* (L.) Hara	Mungei	Whole plant	Herb	Infusion, decoction	[44, 52]

Opiliaceae	*Opilia campestris* Engl.	Enkirashai (Maasai)	Roots	Shrub	Decoction	[22]

Oxalidaceae	*Oxalis corniculata* L.	Nyonyoek (Nandi)	Whole plant	Herb	Decoction	[38]

Papilionaceae	*Cajanus cajan* Millsp.	Mucugu (Kikuyu)	Not specified	Herb	Not specified	[3]
	*Dalbergia lactea* Vatke	Mwaritha (Kikuyu)	Not specified	Shrub	Not specified	[3]
	*Ormocarpum trachycarpum* (Taub.) Harms	Muthingii (Kamba)	Bark, leaves	Shrub	Decoction	[52, 58]
	*Rhynchosia hirta* (Andrews) Meikle & Verdc.	Tilyamook (Nandi)	Roots	Liana	Decoction	[38]
	*Stylosanthes fruticosa* (Retz.) Alston	Kalaa (Kamba)	Leaves, whole plant	Herb	Infusion	[55]

Passifloraceae	*Passiflora ligularis* A. Juss.	Hondo (Kikuyu)	Not specified	Shrub	Not specified	[3]

Piperaceae	*Piper capense* L.f.	Olerrubaat (Maasai)	Roots	Herb	Decoction	[48]

Pittosporaceae	*Pittosporum lanatum* Hutch. & Bruce	Munyamati (Kikuyu)	Not specified	Herb	Not specified	[3]
	*Pittosporum viridiflorum* Sims	Munati	Stem bark	Tree	Decoction	[34, 44, 52]

Poaceae	*Pennisetum hohenackeri* Hochst. ex Steud	Olmakutian (Maasai)	Bark, branches, roots	Grass	Decoction	[67]
	*Rottboellia exaltata* L.f.	Mpunga (Digo)	Leaves	Herb	Decoction	[17, 46]
	*Sporobolus stapfianus*	Not reported	Not specified	Herb	Not specified	[45]

Podocarpaceae	*Podocarpus latifolius* (Thunb.) R.Br. ex Mirb.	Enchani-enkashi (Maasai)	Roots	Tree	Decoction	[48]

Polygonaceae	*Rumex abyssinicus* Jacq.	Shikachi	Leaves	Herb	Decoction	[34]
	*Rumex steudelii* Hochst ex A. Rich	Alukhava	Roots	Herb	Decoction	[34]

Polygalaceae	*Securidaca longifolia* Poepp.	Not reported	Roots	Tree	Decoction	[17]
	*Securidaca longipedunculata* Fres.	Mzigi (Digo)	Roots, bark, leaves	Shrub	Decoction	[46]

Primulaceae	*Myrsine africana* L.	Oseketeki (Maasai)	Seeds, fruits, roots, multiple parts	Shrub	Decoction	[54, 67]

Rahmnaceae	*Rhamnus prinoides* L.'Herit	Orkonyil (Maasai)	Roots, root bark	Shrub	Decoction	[3, 11, 35, 38, 44, 48, 69, 78]
	*Rhamnus staddo* A. Rich	Orkokola (Maasai), Ngukura (Kikuyu)	Root bark, stem bark	Shrub	Decoction	[3, 11, 35, 44, 48, 69]
	*Scutia myrtina* (Burm. f.) Kurz	Osanankoruri (Maasai)	Not specified	Shrub	Not specified	[3]

Ranunculaceae	*Clematis brachiata* Thunb.	Olkisusheeit (Maasai)	Roots, root bark	Liana	Decoction	[44, 48]

Rhizophoraceae	*Cassipourea malosana* (Baker) Alston	Muthathi (Kikuyu)	Not specified	Tree	Not specified	[3]

Rosaceae	*Prunus africana* (Hook. f.) Kalkman	Orkujuk (Maasai), Muiri (Kikuyu)	Bark, root, stem, stem bark	Tree	Decoction	[3, 38, 44, 79, 80]
	*Rubus pinnatus* Wild.	Butunduli	Leaves, bark, fruits	Shrub	Decoction	[3, 34]

Rubiaceae	*Aganthesanthemum bojeri* Klotzsch.	Kahithima	Whole plant	Herb	Not specified	[50]
	*Agathisanthenum globosum* (Hochst. ex A. Rich.) Bremek.	Chivuma nyuchi (Digo)	Roots	Herb	Decoction	[17, 46]
	*Canthium glaucum* Hiern.	Mhonga/Mronga (Digo)	Fruits	Shrub	Decoction	[17, 46]
	*Gardenia ternifolia* subsp. Jovistonatis	Kibulwa	Fruits	Shrub	Decoction	[54]
	*Keetia gueinzii* (Sond.) Bridson	Mugukuma (Kikuyu)	Not specified	Shrub	Not specified	[3]
	*Pentanisia ouranogyne* S. Moore	Chungu (Digo)	Roots	Herb	Decoction	[17, 46]
	*Pentas bussei* K. Krause	Not reported	Root bark	Shrub	Decoction	[17]
	*Pentas longiflora* Oliv.	Muhuha (Kikuyu), Cheroriet (Nandi)	Bark, fruits, leaves, roots	Shrub	Decoction, rub on skin	[3, 17, 38, 41, 61]
	*Pentas lanceolata* (Forssk.) Deflers	Olkilaki-olkerr (Maasai)	Root bark	Herb	Decoction	[48]
	*Rubia cordifolia* L.	Urumurwa (Kuria)	Not specified	Herb	Not specified	[37]
	*Spermacoce princeae* (K. Schum.) Verdc.	Omonhabiebo	Whole plant	Herb	Decoction	[35]
	*Vangueria madagascariensis* Gmel (*Vangueria acutiloba* Robyns)	Mubiru	Stem bark	Shrub	Decoction	[44]
	*Vangueria volkensii* K.Schum.	Kimoluet (Nandi)	Roots	Shrub	Decoction	[38, 47]

Rutaceae	*Citrus aurantiifolia* (Christm.) Swingle	Mutimu (Kikuyu)	Not specified	Tree	Not specified	[3]
	*Citrus limon* (L.) Burm.f.	Ndim (Luo)	Fruits, leaves	Tree	Eaten, decoction	[36]
	*Clausena anisata* (Willd) Hook. f. ex Benth.	Mtondombare (Digo), Mukibia	Leaves, roots, bark, root bark	Shrub	Decoction	[17, 34, 41, 44, 46]
	*Fagaropsis angolensis* (Eng.) H.M. Gardner	Murumu, mukuriampungu	Leaves, roots, stem bark	Tree	Decoction	[3, 23, 38, 44, 53]
	*Fagaropsis angolensis* (Eng.) Dale	Mukaragati (Kikuyu)	Leaves, roots	Tree	Decoction	[3, 17, 46]
	*Fagaropsis hildebrandtii* (Engl.) Milne-Redh.	Muvindavindi (Kamba)	Leaves	Tree	Decoction	[3, 81]
	*Harrisonia abyssinica* Olive	Osiro (Luo), Orongoriwe (Kuria)	Leaves, roots, root bark	Tree	Decoction	[17, 23, 37, 44, 46, 47, 54, 82, 83]
	*Teclea nobilis*	Not reported	Stem bark	Shrub	Decoction	[11, 45]
	*Teclea simplicifolia* (Engl.) Verdoorn	Mutuiu (Kamba), Munderendu (Kikuyu)	Leaves, roots, stem bark	Shrub	Decoction	[3, 17, 44, 46, 55]
	*Toddalia asiatica* (L.) Lam	Mururue (Kikuyu), Oleparmunyo (Maasai)	Roots, root bark, leaves, fruits (multiple parts)	Shrub	Decoction	[3, 11, 17, 44, 45, 47, 58, 59, 62, 67, 84]
	*Zanthoxylum chalybeum* Engl.	Oloisuki (Maasai)	Stem bark, root bark	Tree	Decoction	[3, 17, 44, 46, 55, 61, 71, 85]
	*Zanthoxylum gilletii* (De Wild.) P.G. Waterman	Shihumba/Shikuma	Bark	Tree	Decoction	[34, 86]
	*Zanthoxylum usambarense* (Engl.) Kokwaro	Oloisuki (Maasai)	Root, fruits, bark, leaves, stem	Tree	Decoction	[3, 11, 67, 78, 85]

Salicaceae	*Dovyalis abyssinica* (A. Rich.) Warb	Kaiyaba (Kikuyu)	Leaves, roots	Shrub	Decoction	[3, 38]
	*Dovyalis caffra* (Hook. f. & Harv.) Warb	Mukambura (Kikuyu)	Not specified	Shrub	Not specified	[3]
	*Flacourtia indica* (Burm.f) Merr.	Mtondombare (Digo)	Roots, bark	Shrub	Decoction	[17, 46]
	*Trimeria grandifolia* (Hochst.) Warb	Oledat (Maasai)	Roots	Shrub	Decoction	[3, 38, 47]

Salvadoraceae	*Salvadora persica* L.	Mukayau (Kamba)	Root, stem	Shrub	Decoction; prepared with salt and milk	[22, 51, 63]

Santalaceae	*Osyris lanceolata* Hochst. & Steudel	Olosesiai (Maasai), muthithii (Kikuyu)	Not specified	Shrub	Not specified	[3]

Sapindaceae	*Allophylus pervillei* Blume.	Mvundza kondo	Leaves, roots, bark	Shrub	Decoction	[50]
	*Cardiospermum corundum*	Not reported	Not specified	Shrub	Not specified	[23]
	*Pappea capensis* (Spreng) Eckl. & Zeyh.	Muba (Kikuyu), Enkorr irri (Maasai)	Branches	Shrub	Decoction	[3, 48]

Sapotaceae	*Manilkara butegi*	Anon	Bark	Shrub	Decoction	[54]
	*Mimusops bagshawei* S. Moore	Lolwet (Nandi)	Leaves, bark	Tree	Decoction	[38]

Solanaceae	*Physalis peruviana* L.	Mayengo	Leaves	Shrub	Inhale steam	[34]
	*Solanum aculeastrum* Dunal	Mutura (Kikuyu)	Not specified	Shrub	Not specified	[3]
	*Solanum incanum* L.	Mutongu (Kamba), Entulelei (Maasai)	Roots, leaves, root bark	Shrub	Decoction	[17, 34, 37, 44, 46, 87]
	*Solanum taitense* Vatke	Entemelua (Maasai)	Roots	Shrub	Chewed directly	[22]
	*Withania somnifera* (L.) Dunal	Murumbae (Kikuyu)	Root bark	Shrub	Decoction	[3, 44]

Ulmaceae	*Chaetacme aristate* Planch	Not reported	Roots	Shrub	Decoction	[41]

Urticaceae	*Urtica massaica* Mildbr.	Thabai (Kikuyu)	Aerial parts	Herb	Decoction	[3, 35]

Verbenaceae	*Clerodendrum eriophyllum* Guerke	Muumba	Root bark	Shrub	Decoction	[44, 52]
	*Lantana camara* L.	Ruithiki, Mukenia (Kikuyu)	Leaves	Shrub	Decoction	[3, 73]
	*Lantana trifolia* L.	Ormokongora (Maasai)	Leaves	Shrub	Decoction	[34, 72]
	*Lippia javanica* (Burm.f.) Spreng	Angware-Rao (Luo)	Roots	Herb	Not specified	[37, 58]
	*Premna chrysoclada* (Bojer) Gürke	Mvuma	Roots, leaves	Herb	Not specified	[50]

Vitaceae	*Cissus quinquangularis* L.	Not reported	Not specified	Herb	Not specified	[45]
	*Cyphostemma maranguense* (Gilg) Desc.	Mutambi (Kikuyu)	Not specified	Herb	Not specified	[3]
	*Rhoicissus tridentata* (L.f.) Wild & Drum	Ndurutua (Kikuyu)	Bark, roots	Shrub	Decoction	[3, 34, 38, 62]

Xanthorrhoeaceae	*Aloe deserti* A. Berger	Ngolonje (Digo)	Leaves	Herb	Decoction, infusion	[17, 46]
	*Aloe macrosiphon* Bak.	Golonje (Giriama)	Leaves	Herb	Infusion	[46]
	*Aloe secundiflora* Engl.	Osukuroi (Maasai), Kiluma (Kamba)	Leaves, leaf sap (exudate)	Herb	Infusion, decoction	[11, 17, 34, 43, 44, 46, 58, 78, 88, 89]
	*Aloe vera* (L) Webb.	Alvera (Digo)	Leaves	Herb	Infusion	[17, 46]
	*Rhoicissus revoilli*	Rabongo (Luo)				

Zingiberaceae	*Zingiber officinale*	Tangawizi (Luo)	Roots	Herb	Chewed	[36]

Zygophyllaceae	*Balanites glabrus* Mildbr. & Schltr.	Orng'osua (Maasai)	Not specified	Tree	Not specified	[22]
	*Balanites glabra* Mildbr. & Schltr.	Olng'osua (Maasai)	Bark	Shrub	Decoction	[22]
	*Balanites aegyptiaca* (L.) Del.	Olngosua (Maasai)	Bark	Shrub	Decoction	[48]

*Language* is also known as Kikamba. Local names with language(s) not indicated are sometimes a blend of Kiswahili and other local languages or were not specified by the authors. *Decoction* involves boiling a plant part in water. *Infusion* entails soaking of a plant part in water.

**Table 2:** Side effects, antidotes, and contraindications of medicinal plants used for traditional management of malaria in Kenya.

Plant	Side effects	Antidote(s)	Contraindication	Reference(s)
*Albizia anthelmintica* Brongn.	Induces vomiting, diarrhea, and bile release from the gall bladder	Not reported	Pregnant women	[22]
*Aloe volkensii* L.	Induces vomiting	Not reported	Children	[22]
*Balanites glabrus* Mildbr. & Schltr.	Induces vomiting, diarrhea, and bile release from the gall bladder	Not reported	Pregnant women	[22]
*Croton megalocarpoides* Friis & M.G. Gilbert	Stomachache, induce vomiting, and bile release from the gall bladder	Not reported	Not reported	[22]
*Euphorbia meridionalis* Bally & S. Carter	Induces diarrhea as a means of cleansing the body	Taken with goat or sheep soup	Not reported	[22]
*Momordica friesiorum* Hams C. Jeffrey	Induces vomiting and bile release from the gall bladder	Not reported	Not reported	[54]
*Opilia campestris* Engl.	Induces vomiting and bile release from the gall bladder	Mixed with soup	Not reported	[22]
*Pyrenacantha malvifolia* Engl.	Induces vomiting	Not reported	Pregnant women	[22]
*Salvadora persica* L.	Induces vomiting and bile release	Milk, salt	Not reported	[22]
*Sericocomopsis hildebrandtii* Schinz.	Stomachache, weight loss through induced vomiting, and bile release from the gall bladder	Milk	Pregnant women	[22]

**Table 3:** Antiplasmodial/antimalarial activities of investigated plants used for malaria treatment in Kenya and their active chemical constituents.

Plant	Part used	Extracting solvent	Antiplasmodial (IC_50_ *μ*g/ml)/antimalarial activity (*Plasmodium* strain)	Active phytochemicals and toxicity information	Reference (s)
*Justicia betonica* L.	Shoot	Methanol, water, ether	69.6 (K39), >100 (K39), 13.36 *μ*g/ml	Justetonin (indole(3,2-b) quinoline alkaloid glycoside)	[117, 118]
*Allium sativum* L.	Synthetic	Ethanol	50 mg/kg of ajoene suppressed development of parasitemia; ajoene (50 mg/kg) and chloroquine (4.5 mg/kg), given as a single dose, prevented development of parasitemia	Ajoene, nontoxic	[119]
*Acmella caulirhiza*	Whole plant	Dichloromethane	9.939 (D6); 5.201 (W2)	No reports	[56]
*Aloe kedongensis* Reynolds	Leaves	Methanol	87.7 (D6); 67.8 (W2)	Anthrone, C-glucoside homonataloin, anthraquinones, aloin, lectins	[120, 121]
*Aloe secundiflora* Eng.	Leaf exudate	Tested direct	66.20 (K39)	No reports	[58]
*Achyranthes aspera* L.	Leaf, stem, roots, seeds	Ethanol	>100, 76.75, >100, >100 *μ*g/ml	Alkaloids, glycosides, saponins, triterpenoids	[122]
*Artemisia annua* L.	Leaves	Water	1.1 (D10), 0.9 (W2)	Sesquiterpenes and sesquiterpene lactones including artemisinin; safe and effective; artemisinin is safe for pregnant women	[120, 123, 124]
*Bidens pilosa* L.	Leaves	Dichloromethane, chloroform, water, and methanol	8.5, 5, 11, 70 (D10)	No reports	[76]
*Maytenus undata* (Thunb.) Blakelock	Leaves	Dichloromethane, dichloromethane/chloroform (1 : 1), methanol, water	>100, 21, 60, >100 (D10)	No reports	[76]
	Stem	Dichloromethane, dichloromethane/chloroform (1 : 1), methanol, water	85, 24, 38, >100 (D10)		
	Roots	Dichloromethane, chloroform, methanol, water	23, 36, 40, >100 (D10)		
*Rhus natalensis* Bernh. ex Krauss	Stem bark, leaves	Ethanol	>50 (FcB1)	Triterpenoids	[50, 125]
	Leaves, roots	Methanol	43.92 (D6), 51.2 (W2); >100 (D6), 80.44 (W2)		
*Carissa edulis* (Forssk.) Vahl	Stem bark, root bark, roots	Dichloromethane, chloroform, water, and methanol	33 (D10), 6.41 (D6), >250, 148.53 and >250, >250 against ENT 30, and NF 54, respectively	Lignan, nortrachelogenin, cytotoxicity IC_50_ > 20, LD_50_ of 260.34, and 186.71 *μ*g/ml for water and methanol extracts	[48, 53, 76]
*Euphorbia tirucalli* L.	Leaves	Dichloromethane, dichloromethane/methanol (1 : 1), methanol, water	12, 23.5, >100, 83 (D10)	No reports	[76]
*Psiadia punctulata*	Twigs	Dichloromethane, water	9, >100 (D10)	No reports	[76]
	Leaves	Dichloromethane, dichloromethane/methanol (1 : 1), water	14, 22.5, >100 (D10)		
	Whole plant	Dichloromethane/methanol (1 : 1), water	18 (D10), >100 (D10)		
*Ricinus communis* L.	Leaves	Dichloromethane/methanol (1 : 1), water	27.5, >100 (D10)	No reports	[76]
	Stems	Dichloromethane/methanol (1 : 1), water	8, >100 (D10)		
	Fruit	Dichloromethane/methanol (1 : 1), water	90, >100 (D10)		
*Catharanthus roseus* G. Don	Leaves	Methanol	4.6 (D6); 5.3 (W2)	Has neurotoxic alkaloids, terpenoids, flavonoids, sesquiterpenes	[57, 126]
*Caesalpinia volkensii* Harms	Leaves	Decoction, ethanol, petroleum ether, methanol, water	480, 481, 490, 858, 404 (FCA: 20 GHA), 923, 960, 250, 961, 563 (W2)	No reports	[11]
*Artemisia afra* Jacq. ex Willd	Leaves	Methanol	9.1 (, D6); 3.9 (W2)	Acacetin, genkwanin, 7-methoxyacacetin; cytotoxicity observed in Vero cells	[57, 127]
*Microglossa pyrifolia* (Lam.) O. Ktze	Leaves	Chloroform, dichloromethane	<5 (both NF54 and FCR3)	E-Phytol, 6e-geranylgeraniol-19-oic acid; cytotoxic to human foetal lung fibroblast cell lines	[18, 25, 128, 129]
*Cucumis aculeatus* Cogn	Fruit	Water	>30	No reports	[62]
*Schkuhria pinnata* (Lam.)	Whole plant	Water	22.5 (D6); 51.8 (W2)	Schkuhrin I and schkuhrin II; methanol extract: low cytotoxicity against human cells; aqueous extracts: no toxicity observed in mice	[57, 130]
*Solanecio mannii* (Hook. f.) C. Jeffrey	Leaves	Methanol	21.6 (3D7); 26.2 (W2)	Phytosterols, *n*-alkanes, and *N*-hexacosanol	[120, 128]
*Tagetes minuta* L.	Leaves	Ethyl acetate	61.0% inhibition at 10 *μ*g/ml	No reports	[130]
*Tithonia diversifolia* A. Gray	Leaves, aerial parts	Methanol, ether	1.2 (3D7), 1.5 (W2), methanolic extract had 74% parasitemia suppression	Tagitinin C and sesquiterpene lactones; aerial parts are cytotoxic against cells from the human foetal lung fibroblast cell line.	[128, 131–133]
*Vernonia amygdalina* Del.	Leaves	Methanol/dichloromethane, ethanol	2.7 (K1), 9.83. *In vivo* parasite suppression of between 57.2 and 72.7% in combination with chloroquine	Vernolepin, vernolin, vernolide, vernodalin and hydroxy vernodalin, and steroid glucosides; petroleum ether extract shows strong cytotoxicity	[111, 120, 130, 131, 134, 135]
*Vernonia auriculifera* (Welw.) Hiern	Leaves	Ethane, chloroform, ethyl acetate, water	>100, 37.7, 40.3, 55.2, >100 (K39)	No reports	[35]
*Vernonia brachycalyx* O. Hoffm. Schreber	Leaves	Chloroform/ethyl acetate, methanol	6.6, 31.2 (K39) 29.6, 30.2 (V1/S)	5-Methylcoumarin isomers, 16,17-dihydrobrachycalyxoloid	[58]
*Vernonia lasiopus* O. Hoffm.	Leaves	Methanol	44.3 (D6); 52.4 (W2)	Sesquiterpene lactones, polysaccharides	[57, 120]
*Markhamia lutea* (Benth.) K. Schum.	Leaves	Ethyl acetate	71% inhibition of *P. falciparum* at 10 *μ*g/ml	Phenylpropanoid glycosides, cycloartane triterpenoids, musambins A-C, Candmusambiosides A-C	[130, 136]
*Spathodea campanulata*	Stem bark, leaves	Ethyl acetate, ethanol	28.9% inhibition of *P. falciparum*	Quinone (lapachol)	[130, 137, 138]
*Cassia didymobotrya* Fres.	Leaves	Methanol	23.4 (D6); undetectable (W2)	Alkaloids	[57]
*Warbugia ugandensis* Sprague	Stem bark	Methanol, water	6.4 (D6); 6.9 (W2), 12.9 (D6); 15.6 (W2)	Coloratane sesquiterpenes, e.g., muzigadiolide	[57, 131, 139–141]
		Dichloromethane	69% parasite inhibition		
*Carica papaya* L.	Leaves	Ethyl acetate	2.96 (D10), 3.98 (DD2)	Alkaloids, saponins, tannins, glycosides; no serious toxicity reported; carpaine, an active compound against *P. falciparum* had high selectivity and was nontoxic to normal red blood cells	[142, 143]
*Maytenus senegalensis*	Roots	Ethanol	1.9 (D6), 2.4 (W2)	Terpenoids, pentacyclic triterpenes, e.g., pristimerin; no toxicity observed in ethanol extract	[144, 145]
*Ethulia scheffleri* S.Moore	Leaves	Chloroform/ethyl acetate/methanol	49.8 (K39), 32.2 (V1/S)	No reports	[58]
*Combretum molle* G. Don	Stem bark	Acetone	8.2 (3D7)	Phenolics, punicalagin	[146]
*Momordica foetida* Schumach	Shoot	Water	6.16 (NF54); 0.35 (FCR3)	Saponins, alkaloid, and cardiac glycosides; no pronounced toxicity against human hepatocellular (HepG2) and human urinary bladder carcinoma (ECV-304, derivative of T-24) cells	[25, 134, 147]
*Clutia abyssinica* Jaub. & Spach	Leaves	Methanol	7.8 (D6); 11.3 (W2)	Diterpenes	[57]
*Croton macrostachyus* Olive.	Leaves	Chloroform, dichloromethane	Chemotherapeutic effect of 66–82%, 2 (D6)	Triterpenoids including lupeol	[14, 56]
*Flueggea virosa* (Roxb. ex Willd) Voigt	Leaves	Water/methanol	2.0 (W2)	Bergenin, nontoxic, extracts exposed to murine macrophages did not slow or inhibit growth of cells	[148, 149]
*Erythrina abyssinica* Lam.	Stem bark	Ethyl acetate	83.6% inhibition of *P. falciparum* at 10 *μ*g/ml	Chalcones (5-prenylbutein and homobutein), flavanones including 5-deoxyabyssinin II, abyssinin III, and abyssinone IV	[130, 137]
*Kigelia africana* (Lam.) Benth	Bark, fruit	Chloroform/ethyl acetate, methanol	59.9 (K39), 83.8 (V1/S); fruits had 165.9 (K39)	No reports	[58]
*Trichilia emetica* Vahl	Leaves, twigs	Dichloromethane/methanol (1 : 1)	3.5 for all (D10)	Kurubasch aldehyde	[76, 150]
*Senna didymobotrya* (Fresen.) H. S. Irwin & Barneby	Leaves, twigs	Methanol, dichloromethane/methanol (1 : 1)	>100 (K39), 9.5 (D10)	Quinones	[35, 76, 117]
*Tamarindus indica* L.	Stem bark	Water	25.1% chemosuppressive activity at 10 mg/kg (*P. berghei*)	Saponins (leaves), tannins (fruits)	[73]
*Harungana madagascariensis* Lam.	Stem bark	Water, ethanol	9.64 (K1); <0.5 with 28.6–44.8% parasite suppression	Quinones including bazouanthrone, ferutinin A, harunganin, harunganol A, anthraquinones, saponins, steroids	[137, 151–153]
*Rotheca myricoides* (Hochst.) Steane and Mabb	Leaves	Methanol	9.51–10.56 and 82% parasite suppression at 600 mg/kg	No reports	[154]
*Leucas calostachys* Oliv.	Leaves	Methanol	3.45 with parasite inhibition of 3.5–5.2%	No reports	[82]
*Ajuga remota* Benth.	Whole plant	Ethanol; decoction, ethanol, petroleum ether, methanol, and water	55 (FCA/GHA), 57 (W2); 937, 55, 149, 504, 414 (FCA/GHA), 371, 57, 253, 493, 101 (W2)	Ajugarin-1, ergosterol-5,8-endoperoxide, 8-oacetylharpagide, steroids	[11, 14]
*Suregada zanzibariensis* Baill	Root bark	Water, methanol	≤10 (K67), (ENT36)	Alkaloids	[96, 155]
*Clerodendrum myricoides* R. Br.	Root bark	Ethanol	4.7 (D6); 8.3 (W2)	No reports	[156, 157]
		Chloroform	>10 (D6)	Cytotoxicity, IC_50_ > 20.0 *μ*g/ml	[48]
*Hoslundia opposita* Vahl.	Leaves	Ethyl acetate	66.2% inhibition of *P. falciparum* at 10 *μ*g/ml	Quinones, saponins, abietane diterpenes (3-obenzoylhosloppone)	[50, 130]
	Roots; aerial parts	Methanol	79.38 (D6), 64.21 (W2); 19.73 (D6), 29.41 (W2)		
*Leonotis nepetifolia*	Leaves	Ethyl acetate, dichloromethane/methanol (1 : 1), water	27.0% inhibition of *P. falciparum* at 10 *μ*g/ml, 15, >100 (D10)	No reports	[76, 130]
*Ocimum basilicum L.*	Leaves, whole plant	Ethanol	68.14 (3D7); 67.27 (INDO)	No reports	[156, 157]
*Ocimum gratissimum* Wild	Leaves/twigs	Dichloromethane	8.6 (W2)	Flavonoids	[56, 158]
*Ocimum suave* Wild	Leaves	Water (hot), chloroform/methanol mixture	100 mg/kg/day of extracts provided 81.45% and 78.39% parasite chemosuppression		[71]
*Plectranthus barbatus* Andrews	Leaves	Dichloromethane	No activity	No toxicity recorded	[56, 71]
	Root bark	Water (hot), chloroform/methanol mixture	100 mg/kg/day of extracts had 55.23% and 78.69% parasite chemosuppression		
*Azadirachta indica* A. Juss.	Leaves	Water, methanol	17.9 (D6); 43.7 (W2)	Terpenoids, isoprenoids, gedunin, limonoids: khayanthone, meldenin, and nimbinin; cytotoxicity LD_50_ of 101.26 and 61.43 *μ*g/ml for water and methanol extracts	[53, 144, 158–160]
*Melia azedarach*	Leaves	Methanol, dichloromethane	55.1 (3D7), 19.1 (W2); 28	No reports	[161, 162]
*Ficus thonningii* Blume	Leaves	Hexane	10.4	No reports	[163]
*Cissampelos mucronata* A. Rich.	Root bark, root	Methanol, ethyl acetate	8.8 (D6); 9.2 (W2); root extract <3.91 (D6), 0.24 (W2) for the active compound (curine)	Benzylisoquinoline alkaloids, curine	[74, 75, 157]
*Acacia nilotica* L.	Stem bark	Methanol	100 mg/kg produced 77.7% parasitic inhibition	Tannins, flavonoids, terpenes	[53, 164]
		Water, methanol	>250, 153.79 (ENT 30), 73.59, 70.33 (NF 54)	LD_50_ of 368.11 and 267.31 *μ*g/ml for water and methanol extracts	
*Albizia coriaria* Welw. ex Oliv	Stem bark	Methanol	15.2 (D6); 16.8 (W2)	Triterpenoids, lupeol, lupenone	[57]
*Ageratum conyzoides* L.	Whole plant	Dichloromethane, methanol	2.15 (D6); 3.444 (W2), 11.5 (D6); 12.1 (W2)	Flavonoids	[57]
*Albizia zygia* (DC.) Macbr.	Stem bark	Methanol	1.0 (K1)	Flavonoids mainly 3′,4′,7-trihydroxyflavone; aqueous extract is relatively safe on subacute exposure	[165, 166]
*Maesa lanceolata* Forsk.	Twig	Dichloromethane: methanol (1 : 1)	5.9 (D10)	Lanciaquinones, 2,5, dihydroxy-3-(nonadec-14-enyl)-1,4-benzoquinone	[76, 128, 167]
*Securidaca longipedunculata* Fresen.	Leaves	Dichloromethane	6.9 (D10)	Saponins, flavonoids, alkaloids, steroids	[168]
*Prunus africana* (Hook. f.) Kalkman	Stem bark	Methanol	17.3 (D6); not detected (W2)	Terpenoids	[57]
*Pentas longiflora* Oliv.	Root	Methanol	0.99 (D6); 0.93 (W2)	Pyranonaphthoquinones, pentalongin and psychorubrin, and naphthalene derivative mollugin; low cytotoxicity	[169]
*Teclea nobilis* Delile	Bark	70% ethanol	53.27% suppression of parasitemia at 700 mg/kg	Tannins, alkaloids, saponins, flavonoids	[167, 170]
		Ethyl acetate	54.7% inhibition of *P. falciparum* at 10 *μ*g/ml	Quinoline alkaloids	[130]
*Toddalia asiatica*	Root bark, fruits, and leaves	Methanol, water, ethyl acetate, hexane	6.8 (D6); 13.9 (W2); ethyl acetate fruit extract (1.80 mg/ml), root bark aqueous (2.43) (W2)	Furoquinolines (nitidine and 5,6-dihydronitidine), coumarins; acute and cytotoxicity of the extracts, with the exception of hexane extract from the roots showed LD_50_ > 1000 mg/kg and CC_50_ > 100 mg/ml, respectively	[84, 157]
*Zanthoxylum chalybeum* Engl.	Stem bark	Water	4.3 (NF54); 25.1 (FCR3)	Chelerythine, nitidine, and methyl canadine; no toxicity recorded	[25, 71]
*Trimeria grandifolia* (Hochst.) Warb.	Leaves	Methanol	>50 (3D7)	No reports	[128]
*Harrisonia abyssinica* Olive.	Roots	Water, methanol	4.4 (D6), 10.25 (W2); 89.74, 79.50 (ENT 30); 86.56, 72.66 (NF 54)	Limonoids and steroids; LD_50_ of 234.71 and 217.34 *μ*g/ml for water and methanol extracts	[53, 144]
*Lantana camara* L.	Leaves, leaves/twigs	Dichloromethane, dichloromethane/methanol (1 : 1), water	8.7 (3D7), 5.7 (W2), 11 (D10), >100 (D10), >100 (D10)	Lantanine, sesquiterpenes, triterpenes, flavonoids	[76, 171]
*Flacourtia indica* (Burm. f.) Merr.	Roots	Dichloromethane, dichloromethane/methanol (1 : 1), water	86.5 (D10), 78 (D10), >100 (D10)	No reports	[76]
*Clausena anisata*	Twigs, leaves	Dichloromethane/methanol (1 : 1), water	18 (D10), >100 (D10); 55, >100 (D10)	No reports	[76]
*Flueggea virosa* (Roxb.ex Willd.) Baill.	Leaves/twigs	Dichloromethane/methanol (1 : 1), water	19 (D10), 11.4 (D10) Alkaloids: Securinine and viroallosecurinine had IC_50_ of 2.7 and 2.9	Alkaloids, bergenin (root bark), securinine, and viroallosecurinine	[76, 172–174]
*Lantana trifolia* L.	Ariel parts	Petroleum ether, chloroform, ethanol	13.2, >50, >50 (plasmodial lactate dehydrogenase)	Steroids, terpenoids, alkaloids, saponins	[125]
*Bridelia micrantha* (Hochst.) Baill.	Stem bark, leaves	Methanol	158.7 (K1)	No reports	[175]
*Balanites aegyptiaca* (L.) Del.	Root bark	Chloroform	3.49 (D6)	Cytotoxicity IC_50_ > 20 *μ*g/ml	[48]
*Sericocomopsis hildebrandtii*	Root bark	Chloroform	3.78 (D6)	Cytotoxicity IC_50_ > 20 *μ*g/ml	[48]
*Boscia angustifolia*	Inner bark	Chloroform	>10.0 (D6); not active	Cytotoxicity IC_50_ > 20 *μ*g/ml	[48]
*Acacia tortilis*	Root bark	Chloroform	>10.0 (D6); not active	Cytotoxicity IC_50_ > 20 *μ*g/ml	[48]
*Commiphora schimperi*	Inner bark	Chloroform	4.63 (D6)	Cytotoxicity IC_50_ > 20 *μ*g/ml	[48]
*Acacia mellifera*	Inner bark	Chloroform	4.48 (D6)	Cytotoxicity IC_50_ > 20 *μ*g/ml	[48]
*Fuerstia africana*	Leaf, aerial parts, leaves	Chloroform, petroleum ether, methanol	3.76 (D6), 1.5, <15 with >70% parasite suppression	Ferruginol, cytotoxicity IC_50_ > 20 *μ*g/ml	[48, 65, 131, 176]
*Psiadia punctulata*	Root bark	Chloroform	>10.0 (D6); not active	Cytotoxicity IC_50_ > 20 *μ*g/ml	[48]
*Ajuga integrifolia* Buch.-Ham	Leaves	Methanol	35.17% at 800 mg/kg/day parasite suppression	Alkaloids, flavonoids, saponins, terpenoids, anthraquinone, steroids, tannins, phenols, and fatty acids; no lethal effect on mice in 24 h and within 10 days of observation	[177]
*Albizia gummifera*		Methanol	0.16 (NF54), 0.99 (ENT 30) for alkaloidal fraction, spermine alkaloids had parasite suppression of 43–72%	Spermine alkaloids (budmunchiamine K, 6-hydroxybudmunchiamine K, 5-normethylbudmunchiamine K, 6-hydroxy-5-normethylbudmunchiamine K, 9-normethylbudmunchiamine K)	[178]
*Rhamnus staddo*	Root bark	Chloroform	>10.0 (D6); not active	Cytotoxicity IC_50_ > 20 *μ*g/ml	[48]
*Ocimum kilimandscharicum*	Leaves, twigs	Dichloromethane	0.843 (D6); 1.547 (W2)	No reports	[56]
*Gutenbergia cordifolia*	Leaves	Chloroform	0.4 (D6)	Cytotoxicity IC_50_ = 0.2 *μ*g/ml	[48]
*Piper capense*	Root bark	Chloroform	>10.0 (D6); not active	Cytotoxicity IC_50_ > 20 *μ*g/ml	[48]
*Pentas lanceolata*	Root bark	Chloroform	5.15 (D6)	Cytotoxicity IC_50_ > 20 *μ*g/ml	[48]
*Clematis brachiata*	Root bark	Chloroform	4.15 (D6)	Cytotoxicity IC_50_ > 20 *μ*g/ml	[48]
*Ekebergia capensis*	Inner bark, fruit, twigs	Chloroform, dichloromethane/methanol (1 : 1)	3.97 (D6), 10, 18 (D10)	Cytotoxicity IC_50_ > 20 *μ*g/ml	[48, 76]
*Rhamnus prinoides*	Root bark	Chloroform	3.53 (D6)	Cytotoxicity IC_50_ > 20 *μ*g/ml	[48]
*Olea europaea* ssp. Africana	Inner bark, leaves, twigs	Chloroform, dichloromethane/methanol (1 : 1)	9.48 (D6), 12, 13 (D10)	Cytotoxicity IC_50_ > 20 *μ*g/ml	[48, 76]
*Pappea capensis*	Inner bark	Chloroform	>10.0 (D6); not active	Cytotoxicity IC_50_ > 20 *μ*g/ml	[48]
*Pittosporum viridiflorum* Sims	Whole plant, leaves/flowers	Dichloromethane, methanol, dichloromethane/methanol (1 : 1)	3, 10, 27.7, (D10), 28, 47, 70.5 (D10)	Triterpenoid estersaponin, pittoviridoside (saponins)	[76, 179, 180]
*Podocarpus latifolius*	Root bark	Chloroform	6.43 (D6)	Cytotoxicity IC_50_ > 20 *μ*g/ml	[48]
*Rumex abyssinicus* Jacq.	Root	Dichloromethane	<15	No reports	[176]
*Rubus pinnatus* Wild	Leaves	Ethanol	20% parasite suppression	No reports	[130]
*Zanthoxylum gilletii*	Stem bark	Dichloromethane/methanol (1 : 1)	2.52 (W2), 1.48 (D6), 1.43 (3D7)	Nitidine, seas amine 8-acetyl dihydrochelerythrine	[86, 176]
*Solanum incanum* L.	Leaves	Chloroform/methanol	31% parasite suppression	No reports	[87]
*Rhoicissus tridentata*	Roots	Water	>40.0	No reports	[62]
*Acacia hockii*	Root bark	Chloroform	>10.0 (D6); not active	Cytotoxicity IC_50_ > 20 *μ*g/ml	[48]
*Lippia javanica* (Burm.f.) Spreng	Roots	Chloroform/ethyl acetate, methanol	16.7, 40.6 (K39), 19.2, 40.1 (V1/S)	No reports	[58, 76]
	Roots, stem	Dichloromethane, methanol, dichloromethane/methanol (1 : 1)	3.8, 27, 24 (D10), 4.5, 21.8, 29.8 (D10)		
*Premna chrysoclada* (Bojer) Gürke	Roots, leaves	Methanol	27.63 (D6), 52.35 (W2); 7.75 (D6), 9.02 (W2)	Not cytotoxic at 100 *μ*g/ml	[50]
*Allophylus pervillei* Blume	Roots, stem bark	Methanol	45.62 (D6), 48.91 (W2); >100 (D6),>100 (W2)	Not cytotoxic at 100 *μ*g/ml	[50]
*Aganthesanthemum bojeri* Klotzsch.	Whole plant	Methanol	55.3 (D6), 55.97 (W2)	Not cytotoxic at 100 *μ*g/ml	[50]
*Abrus precatorius* L.	Leaves	Methanol	85.59 (D6), >100 (W2)	Not cytotoxic at 100 *μ*g/ml	[50]
*Combretum illairii* Engl.	Stem bark, leaves	Methanol	55.96 (D6), 58.54 (W2); 24.21 (D6), 33.31 (W2)	Not cytotoxic at 100 *μ*g/ml	[50]
*Grewia plagiophylla* K. Schum	Leaves, stem bark	Methanol	13.28 (D6), 34.2 (W2); >100 (D6), >100 (W2)	Not cytotoxic at 100 *μ*g/ml	[50]
*Combretum padoides* Engl. & Diels	Roots	Methanol	21.73 (D6), 59.43 (W2)	Not cytotoxic at 100 *μ*g/ml	[50]
*Uvaria acuminata*	Leaves, roots	Methanol	51.13 (D6), >100 (W2); 8.89 (D6), 6.90 (W2)	Cytotoxic with CC_50_ of 2.37 *μ*g/ml.	[50]
*Ormocarpum trachycarpum*	Roots	Chloroform/ethyl acetate, methanol, water	19.6, 41.7, 79.4 (K39); 17.5, 32.8 (V1/S)	No reports	[58]
*Plectranthus sylvestris* Gurke	Leaves	Chloroform/ethyl acetate, methanol	41.1, 56.2 (K39); 61.0 (V1/S)	No reports	[58]
*Turraea robusta*	Root bark	Water, methanol	25.32, 2.09 (D6), 42.41, 10.32 (W2)	IC_50_ of 24.38 and 45.72 *μ*g/ml for methanol and aqueous extracts against Vero cells (cytotoxic)	[49]
*Lannea schweinfurthii*	Stem bark	Water, methanol	10.55 and 75.90, 11.38 and 36.26 (D6 and W2)	IC_50_ of 225.25 and 3256.52 *μ*g/ml for methanol and aqueous extracts against Vero cells	[49]
*Sclerocarya birrea*	Stem bark	Water, methanol	18.96 and 71.74, 5.91 and 24.96 (D6 and W2)	IC_50_ of 361.24 and 3375.22 *μ*g/ml for methanol and aqueous extracts against Vero cells	[49]
*Withania somnifera*	Stem bark	Water, methanol	>250, >250 (ENT 30); 145.86, 125.59 (NF 54)	LD_50_ of 301.44 and 207.27 *μ*g/ml for water and methanol extracts	[53]
*Zanthoxylum usambarense*	Stem bark	Water, methanol	14.33, 5.25 (ENT 30); 5.54, 3.20 (NF 54)	LD_50_ of 260.90 and 97.66 *μ*g/ml for water and methanol extracts	[53]
*Fagaropsis angolensis*	Stem bark	Water, methanol	10.65, 6.13 (ENT 30); 5.04, 4.68 (NF 54)	LD_50_ of 173.48 and 57.09 *μ*g/ml for water and methanol extracts	[53]
*Myrica salicifolia*	Stem bark	Water, methanol	85.97, 66.84 (ENT 30); 55.89, 51.07 (NF 54)	LD_50_ of 328.22 and 320.17 *μ*g/ml for water and methanol extracts	[53]
*Strychnos henningsii* Gilg	Stem bark	Water, methanol	73.39, 67.16 (ENT 30); 190.0, 159.71 (NF 54)	LD_50_ of 293.93 and 101.22 *μ*g/ml for water and methanol extracts	[53]
*Neoboutonia macrocalyx*	Stem bark	Water, methanol	92.85, 84.56 (ENT 30); 78.44, 78.40 (NF 54)	LD_50_ of 41.69 and 21.04 *μ*g/ml for water and methanol extracts	[53]
*Urtica massaica* Mildbr.	Aerial parts	Hexane, chloroform, ethyl acetate, water, methanol	>100 (K39)	No reports	[35]
*Uvaria scheffleri* Diels	Leaves, stem, root bark	Petroleum ether, dichloromethane, methanol	5–500 (K1)	Indole alkaloid-(±L)-schefflone, uvaretin, diuvaretin	[181, 182]
*Rauwolfia cothen*	Root bark	Petroleum ether, dichloromethane, methanol	0–499 (K1)	Yohimbine, an indole alkaloid	[183, 184]
*Tridax procumbens L.*	Whole plant	Dichloromethane/methanol (1 : 1), water	17 (D10), >100 (D10)	Bergenin	[76, 184, 185]
*Centella asiatica*	Leaves	Dichloromethane/methanol (1 : 1)	8.3 (D10)	Alkaloids, sesquiterpenes	[76, 186]
*Ficus sur*	Stem bark	Hexane, chloroform, ethyl acetate, water, methanol	19.2, 9.0, >100, >100, >100 (K39)	No reports	[35]
*Euphorbia inaequilatera* Sond.	Whole plant	Hexane, chloroform, ethyl acetate, water, methanol	19.2, 9.0, >100, >100, >100 (K39)	No reports	[35]
*Spermacoce princeae* (K. Schum.) Verdc.	Whole plant	Hexane, chloroform, ethyl acetate, water, methanol	>100 (K39)	No reports	[35]
*Senna occidentalis*	Leaves	Dimethyl sulfoxide, ethanol	48.80 (3D7), 54.28 (NIDO); <3;	Quinones	[156, 187, 188]
		Ethanol, dichloromethane	>60% parasitemia suppression		
*Searsia natalensis* (Bernh. ex C. Krauss)	Leaves	Chloroform	1.8 (plasmodial lactate dehydrogenase)	No reports	[125]

*Plasmodium falciparum* isolates: D6, 3D7, D10, FCA/GHA (FCA: 20 GHA), FCR3, K39, and NF54 are chloroquine sensitive; DD2, ENT 30, FCR3, K1, NIDO, V1/S, and W2 are chloroquine resistant. For [48], control used for cytotoxicity study (vinblastine) had the effective dose to inhibit 50% growth (ED_50_) = 0.038 *μ*g/ml. An ED_50_ greater than 20 *μ*g/ml indicates that the plant extract lacks cytotoxicity. The control drug chloroquine had a toxicity of 17.4 *μ*g/ml and IC_50_ of 0.004 *μ*g/ml against D6 clone.

## Data Availability

This is a review article, and no raw data were generated. All data generated or analyzed in this study are included in this article.

## References

[1] WHO (2011). *World Malaria Report*.

[2] Burness Communications (21 April 2011). Antimalarial trees in East Africa threatened with extinction. *ScienceDaily*.

[3] Njoroge G. N., Bussman R. W. (2006). Diversity and utilization of antimalarial ethnophytotherapeutic remedies among the Kikuyus (Central Kenya). *Journal of Ethnobiology and Ethnomedicine*.

[4] World Health Organization (2000). *World Health Report 2000*.

[5] World Health Organization (2017). *World Malaria Report 2017*.

[6] President’s Malaria Initiative, Kenya, Malaria Operational Plan FY, 2018, https://www.pmi.gov/docs/default-source/default-document-library/malaria-operational-plans/fy-2018/fy-2018-kenya-malaria-operational-plan.pdf?sfvrsn=5

[7] Machini B., Waqo E., Kizito W. (2016). Trends in outpatient malaria cases, following Mass Long Lasting Insecticidal Nets (LLIN) distribution in epidemic prone and endemic areas of Kenya. *East African Medical Journal*.

[8] Murphy M. W., Dunton R. F., Perich M. J., Rowley W. A. (2001). Attraction of Anopheles (Diptera: Culicidae) to volatile chemicals in Western Kenya. *Journal of Medical Entomology*.

[9] Milliken W. (1997). Traditional anti-malarial medicine in Roraima, Brazil. *Economic Botany*.

[10] Dianne J., Jeanne M., Margarette S. (2003). Treatment history and treatment dose are important determinants of sulfadoxine-pyrimethamine efficacy in children with uncomplicated malaria in Western Kenya. *Journal of Infectious Diseases*.

[11] Kuria K. A. M., De Coster S., Muriuki G. (2001). Antimalarial activity of Ajuga remota Benth (Labiatae) and Caesalpinia volkensii Harms (Caesalpiniaceae): in vitro confirmation of ethnopharmacological use. *Journal of Ethnopharmacology*.

[12] White N. J. (2007). Cardiotoxicity of antimalarial drugs. *The Lancet Infectious Diseases*.

[13] Price R. N., Uhlemann A.-C., Brockman A. (2004). Mefloquine resistance in Plasmodium falciparum and increased pfmdr1 gene copy number. *The Lancet*.

[14] Rahmatullah M., Hossan S., Khatun A., Seraj S., Jahan R. (2012). Medicinal plants used by various tribes of Bangladesh for treatment of malaria. *Malaria Research and Treatment*.

[15] Division of Malaria Control (Ministry of Public Health and Sanitation), Kenya National Bureau of Statistics and ICF Macro 2011; 2010 Kenya Malaria Indicator Survey, DO MC, KNBS and ICF Macro, Nairobi, Kenya

[16] Helen L. G., Sarah K. C., Timothy P. R. (2002). Malaria prevention in highland Kenya: indoor residual house spraying vs. insecticide-treated bed nets. *Tropical Medicine and International Health*.

[17] Nguta J. M., Mbaria J. M., Gathumbi P. K. (2011). Ethnodiagnostic skills of the Digo community for malaria: a lead to traditional bioprospecting. *Frontiers in Pharmacology*.

[18] Pan W.-H., Xu X.-Y., Shi N., Tsang S., Zhang H.-J. (2018). Antimalarial activity of plant metabolites. *International Journal of Molecular Sciences*.

[19] Omara T., Kiprop A. K., Ramkat R. C. (2020). Medicinal plants used in traditional management of cancer in Uganda: a review of ethnobotanical surveys, phytochemistry, and anticancer studies. *Evidence-Based Complementary and Alternative Medicine*.

[20] Omara T., Kagoya S., Openy A. (2020). Antivenin plants used for treatment of snakebites in Uganda: ethnobotanical reports and pharmacological evidences. *Tropical Medicine and Health*.

[21] Bongomin O., Ocen G. G., Nganyi E. O., Musinguzi A., Omara T. (2020). Exponential disruptive technologies and the required skills of Industry 4.0. *Journal of Engineering*.

[22] Kiringe J. W. (2006). A survey of traditional health remedies used by the Maasai of Southern Kaijiado district, Kenya. *Ethnobotany Research and Applications*.

[23] Kareru P. G., Kenji G. M., Gachanja A. N., Keriko J. M., Mungai G. (2007). Traditional medicines among the Embu and Mbeere people of Kenya. *African Journal of Traditional, Complementary and Alternative Medicine*.

[24] Samuelsson G., Farah M. H., Claeson P. (1992). Inventory of plants used in traditional medicine in Somalia. III. Plants of the families Lauraceae-Papilionaceae. *Journal of Ethnopharmacology*.

[25] Adia M. M., Emami S. N., Byamukama R., Faye I., Borg-Karlson A.-K. (2016). Antiplasmodial activity and phytochemical analysis of extracts from selected Ugandan medicinal plants. *Journal of Ethnopharmacology*.

[26] Okello D., Kang Y. (2019). Exploring antimalarial herbal plants across communities in Uganda based on electronic data. *Evidence-Based Complementary and Alternative Medicine*.

[27] Pierre S., Toua V., Tchobsala, Fohouo T. (2011). Medicinal plants used in traditional treatment of malaria in Cameroon. *Journal of Ecology and the Natural Environment*.

[28] Ngarivhume T., van’t Klooster C. I. E. A., de Jong J. T. V. M., Van der Westhuizen J. H. (2015). Medicinal plants used by traditional healers for the treatment of malaria in the Chipinge district in Zimbabwe. *Journal of Ethnopharmacology*.

[29] Chhabra S. C., Mahunnah R. L. A., Mshiu E. N. (1993). Plants used in traditional medicine in eastern Tanzania. VI. Angiosperms (Sapotaceae to Zingiberaceae). *Journal of Ethnopharmacology*.

[30] Watt J. M., Breyer-Brandwijk M. G. (1962). *The Medicinal and Poisonous Plants of Southern and Eastern Africa*.

[31] Eneyew A., Asfaw Z., Kelbessa E., Nagappan R. (2014). Ethnobotanical study of traditional medicinal plants in and around Fiche district, Central Ethiopia. *Current Research Journal of Biological Sciences*.

[32] Taek M., Prajogo B., Agil M. (2018). Plants used in traditional medicine for treatment of malaria by Tetun ethnic people in West Timor Indonesia. *Asian Pacific Journal of Tropical Medicine*.

[33] Alebie G., Urga B., Worku A. (2017). Systematic review on traditional medicinal plants used for the treatment of malaria in Ethiopia: trends and perspectives. *Malaria Journal*.

[34] Mukungu N., Abuga K., Okalebo F., Ingwela R., Mwangi J. (2016). Medicinal plants used for management of malaria among the Luhya community of Kakamega East sub-county, Kenya. *Journal of Ethnopharmacology*.

[35] Muregi F. W., Chbabra S. C., Njagi E. N. (2003). In vitro antiplasmodial activity of some plants used in Kisii, Kenya against malaria and their chloroquine potentiation effects. *Journal of Ethnopharmacology*.

[36] Nagata J. M., Jew A. R., Kimeu J. M., Salmen C. R., Bukusi E. A., Cohen C. R. (2011). Medical pluralism on Mfangano island: use of medicinal plants among persons living with HIV/AIDS in Suba district, Kenya. *Journal of Ethnopharmacology*.

[37] Nyambati G. K., Maranga R. O., Ozwara H., Mbugua P. K. (2018). Use of putative antimalarial herbal medicines among communities in Trans-Mara, Kuria and Suba districts of Kenya. *SEJ Pharmacognosy*.

[38] Jeruto P., Mutai C., Ouma G., Lukhoba C. (2011). An inventory of medicinal plants that the people of Nandi use to treat malaria. *Journal of Animal & Plant Sciences*.

[39] Kigen G., Some F., Kibosia J. (2014). Ethnomedicinal plants traditionally used by the Keiyo community in Elgeyo Marakwet county, Kenya. *Journal of Biodiversity, Bioprospecting and Development*.

[40] Jeruto P., Lukhoba C., Ouma G., Otieno D., Mutai C. (2008). An ethnobotanical study of medicinal plants used by the Nandi people in Kenya. *Journal of Ethnopharmacology*.

[41] Okello S. V., Nyunja R. O., Netondo G. W., Onyango J. C. (2010). Ethnobotanical study of medicinal plants used by Sabaots of Mt. Elgon Kenya. *African Journal of Traditional, Complementary and Alternative Medicine*.

[42] Olala C. N. (2014). Identification of plants used for treatment of malaria and factors influencing their use in Boro division, Siaya county, Kenya.

[43] Onyambu M. O., Gikonyo N. K., Nyambaka H. N., Thoithi G. N. (2019). A review of trends in herbal drugs standardization, regulation and integration to the national healthcare systems in Kenya and the globe. *International Journal of Pharmacognosy and Chinese Medicine*.

[44] Muthaura C. N., Rukunga G. M., Chhabra S. C., Mungai G. M., Njagi E. N. M. (2007). Traditional phytotherapy of some remedies used in treatment of malaria in Meru district of Kenya. *South African Journal of Botany*.

[45] Bussmann R. W., Gilbreath G. G., Solio J. (2006). Plant use of the Maasai of Sekenani valley, Maasai Mara, Kenya. *Journal of Ethnobiology and Ethnomedicine*.

[46] Nguta J. M., Mbaria J. M., Gakuya D. W., Gathumbi P. K., Kiama S. G. (2010). Traditional antimalarial phytotherapy remedies used by the South Coast community, Kenya. *Journal of Ethnopharmacology*.

[47] Orwa J. A., Mwitari P. G., Mtu E., Rukunga G. M. (2008). Traditional healers and the management of Malaria in Kisumu district, Kenya. *East African Medical Journal*.

[48] Koch A., Tamez P., Pezzuto J., Soejarto D. (2005). Evaluation of plants used for antimalarial treatment by the Maasai of Kenya. *Journal of Ethnopharmacology*.

[49] Gathirwa J. W., Rukunga G. M., Njagi E. N. M. (2008). The in vitro anti-plasmodial and in vivo anti-malarial efficacy of combinations of some medicinal plants used traditionally for treatment of malaria by the Meru community in Kenya. *Journal of Ethnopharmacology*.

[50] Gathirwa J. W., Rukunga G. M., Mwitari P. G. (2011). Traditional herbal antimalarial therapy in Kilifi district, Kenya. *Journal of Ethnopharmacology*.

[51] Kimondo J., Miaron J., Mutai P., Njogu P. (2015). Ethnobotanical survey of food and medicinal plants of the Ilkisonko Maasai community in Kenya. *Journal of Ethnopharmacology*.

[52] Kokwaro J. O. (1993). *Medicinal Plants of East Africa*.

[53] Kirira P. G., Rukunga G. M., Wanyonyi A. W. (2006). Anti-plasmodial activity and toxicity of extracts of plants used in traditional malaria therapy in Meru and Kilifi districts of Kenya. *Journal of Ethnopharmacology*.

[54] Kaendi J. M. (1994). *Coping with Malaria and Visceral Leishmaniasis (Kala-azar) in Baringo District, Kenya: Implications for Disease Control*.

[55] Kisangau D., Kauti M., Mwobobia R., Kanui T., Musimba N. (2017). Traditional knowledge on use of medicinal plants in Kitui county, Kenya. *International Journal of Ethnobiology and Ethnomedicine*.

[56] Owuor B. O., Ochanda J. O., Kokwaro J. O. (2012). In vitro antiplasmodial activity of selected Luo and Kuria medicinal plants. *Journal of Ethnopharmacology*.

[57] Muthaura C. N., Keriko J. M., Mutai C. (2015). Antiplasmodial potential of traditional phytotherapy of some remedies used in treatment of malaria in Meru-Tharaka Nithi county of Kenya. *Journal of Ethnopharmacology*.

[58] Oketch-Rabah H. A., Dossaji S. F., Mberu E. K. (1999). Antimalarial activity of some Kenyan medicinal plants. *Pharmaceutical Biology*.

[59] Kigen G., Kamuren Z., Njiru E., Wanjohi B., Kipkore W. (2019). Ethnomedical survey of the plants used by traditional healers in Narok county, Kenya. *Evidence-Based Complementary and Alternative Medicine*.

[60] Prota4U *Combretum Padoides Engl. & Diels*.

[61] Kipkore W., Wanjohi B., Rono H., Kigen G. (2014). A study of the medicinal plants used by the Marakwet community in Kenya. *Journal of Ethnobiology and Ethnomedicine*.

[62] Gakunju D. M., Mberu E. K., Dossaji S. F. (1995). Potent antimalarial activity of the alkaloid nitidine, isolated from a Kenyan herbal remedy. *Antimicrobial Agents and Chemotherapy*.

[63] Nankaya J., Gichuki N., Lukhoba C., Balslev H. (2019). Medicinal plants of the Maasai of Kenya: a review. *Plants*.

[64] Omwenga E. O., Hensel A., Shitandi A., Goycoolea F. M. (2015). Ethnobotanical survey of traditionally used medicinal plants for infections of skin, gastrointestinal tract, urinary tract and the oral cavity in Borabu sub-county, Nyamira county, Kenya. *Journal of Ethnopharmacology*.

[65] Kigondu E. V. M., Matu E. N., Gathirwa J. W. (2011). Medicinal properties of Fuerstia africana T.C.E. Friers (Lamiaceae). *African Journal of Health Sciences*.

[66] Rukunga G. M., Gathirwa J. W., Omar S. A. (2009). Anti-plasmodial activity of the extracts of some Kenyan medicinal plants. *Journal of Ethnopharmacology*.

[67] Nankaya J., Nampushi J., Petenya S., Balslev H. (2019). Ethnomedicinal plants of the Loita Maasai of Kenya. *Environment, Development and Sustainability*.

[68] Kuria K. A. M., Chepkwony H., Govaerts C. (2002). The antiplasmodial activity of isolates from Ajugaremota. *Journal of Natural Products*.

[69] Muregi F. W., Ishih A., Miyase T. (2007). Antimalarial activity of methanolic extracts from plants used in Kenyan ethnomedicine and their interactions with chloroquine (CQ) against a CQ-tolerant rodent parasite, in mice. *Journal of Ethnopharmacology*.

[70] Okach D. O., Nyunja A. R. O., Opande G. (2013). Phytochemical screening of some wild plants from Lamiaceae and their role in traditional medicine in Uriri district–Kenya. *International Journal of Herbal Medicine*.

[71] Kiraithe M. N., Nguta J. M., Mbaria J. M., Kiama S. G. (2016). Evaluation of the use of Ocimum suave Willd. (Lamiaceae), Plectranthus barbatus Andrews (Lamiaceae) and Zanthoxylum chalybeum Engl. (Rutaceae) as antimalarial remedies in Kenyan folk medicine. *Journal of Ethnopharmacology*.

[72] Otieno N. E., Analo C. (2012). Local indigenous knowledge about some medicinal plants in and around Kakamega forest in Western Kenya. *F1000Research*.

[73] Nguta J. M., Mbaria J. M. (2013). Brine shrimp toxicity and antimalarial activity of some plants traditionally used in treatment of malaria in Msambweni district of Kenya. *Journal of Ethnopharmacology*.

[74] Omole R. A. (2012). Anti-malarial activity and phytochemical studies of Cissampelos mucronata and Stephania abyssinica.

[75] Ndiege I. O. (2011). Anti-malarial activity and phytochemical studies of Cissampelos mucronata and Stephania abyssinica.

[76] Clarkson C., Maharaj V. J., Crouch N. R. (2004). In vitro antiplasmodial activity of medicinal plants native to or naturalised in South Africa. *Journal of Ethnopharmacology*.

[77] Kemboi D. (2016). Review of traditionally used medicinal plants by the Kipsigis community in Kenya. *British Journal of Pharmaceutical Research*.

[78] Muthee J. K., Gakuya D. W., Mbaria J. M., Kareru P. G., Mulei C. M., Njonge F. K. (2011). Ethnobotanical study of anthelmintic and other medicinal plants traditionally used in Loitoktok district of Kenya. *Journal of Ethnopharmacology*.

[79] Nyamai D. W., Mawia A. M., Wambua F. K. (2015). Phytochemical profile of Prunus africana stem bark from Kenya. *Journal of Pharmacognosy and Natural Products*.

[80] Amuka O., Machocho A. K., Mbugua P. K., Okemo P. O. (2014). Ethnobotanical survey of selected medicinal plants used by the Ogiek communities in Kenya against microbial infections. *Ethnobotany Research and Applications*.

[81] Maundu P., Tengnèas B., Birnie A., Muema N. (2005). *Useful Trees and Shrubs for Kenya*.

[82] Nyambati G. K., Lagat Z. O., Maranga R. O., Samuel M. I., Ozwara H. (2013). In vitro anti-plasmodial activity of Rubia cordifolia, Harrizonia abyssinica, Leucas calostachys Oliv and Sanchus schweinfurthii medicinal plants. *Journal of Applied Pharmaceutical Science*.

[83] Nyambati G. K., Lagat Z. O., Maranga R. O., Samuel M. I., Ozwara H. (2015). Anti-plasmodial activity and toxicity of selected crude plant extracts from Kenya, against Plasmodium berghei in Balb/C mice. *International Journal of Current Research*.

[84] Orwa J. A., Ngeny L., Mwikwabe N. M., Ondicho J., Jondiko I. J. O. (2013). Antimalarial and safety evaluation of extracts from *Toddalia asiatica* (L) Lam. (Rutaceae). *Journal of Ethnopharmacology*.

[85] Kato A., Moriyasu M., Ichimaru M. (1996). Isolation of alkaloidal constituents of Zanthoxylum usambarense and Zanthoxylum chalybeum using ion-pair HPLC. *Journal of Natural Products*.

[86] Masinde W. R. G. (2014). *Phytochemical Investigation of Zanthoxylum Gilletii (Rutaceae) for Antiplasmodial Biomolecules Chemistry*.

[87] Murithi C., Fidahusein D., Nguta J., Lukhoba C. (2014). Antimalarial activity and in vivo toxicity of selected medicinal plants naturalized in Kenya. *International Journal of Education Research*.

[88] Kokwaro J. O. (2009). *Medicinal Plants of East Africa*.

[89] Kamau L. N., Mbaabu P. M., Karuri P. G., Mbaria J. M., Kiama S. G. (2017). Medicinal plants used in the management of diabetes by traditional healers of Narok county, Kenya. *Tang Medicine*.

[90] Maroyi A. (2013). Traditional use of medicinal plants in south-central Zimbabwe: review and perspectives. *Journal of Ethnobiology and Ethnomedicine*.

[91] Musa S. M., Abdelrasool F. E., Elsheikh A. E. (2011). Ethnobotanical study of medicinal plants in the Blue nile state, south-eastern Sudan. *Journal of Medicinal Plants Research*.

[92] Adekunle M. F. (2008). Indigenous uses of plant leaves to treat malaria fever at Omo Forest reserve (OFR) Ogun state, Nigeria. *Ethiopian Journal of Environmental Studies and Management*.

[93] Teklay A., Abera B., Giday M. (2013). An ethnobotanical study of medicinal plants used in Kilte Awulaelo district, Tigray region of Ethiopia. *Journal of Ethnobiology and Ethnomedicine*.

[94] Sindiga I. (1994). Indigenous medical knowledge of the Maasai. *Indigenous Knowledge and Development Monitor*.

[95] Gessler M. C., Nkunya M. H. H., Mwasumbi M., Heinrich M., Tanner M. (1994). Screening Tanzanian medicinal plants for antimalarial activity. *Acta Tropica*.

[96] Omulokoli E., Khan B., Chhabra S. C. (1997). Antiplasmodial activity of four Kenyan medicinal plants. *Journal of Ethnopharmacology*.

[97] Parida M. M., Upadhyay C., Pandya G., Jana A. M. (2002). Inhibitory potential of neem (*Azadirachta indica* Juss) leaves on Dengue virus type-2 replication. *Journal of Ethnopharmacology*.

[98] Neuwinger H. D. (1996). *African Ethnobotany: Poisons and Drugs: Chemistry, Pharmacology, Toxicology*.

[99] Nobili S., Lippi D., Witort E. (2009). Natural compounds for cancer treatment and prevention. *Pharmacological Research*.

[100] Jordan M. A., Wilson L. (2004). Microtubules as a target for anticancer drugs. *Nature Reviews Cancer*.

[101] Annan K., Dickson R. (2008). Evaluation of wound healing actions of Hoslundia opposita Vahl, Anthocleista nobilis G. Don. and *Balanites aegyptiaca* L. *Journal of Science and Technology*.

[102] Sherines R. J., Howard S. S., Harrison J. H., Gittes R. F., Perlmutter A. D., Stamey T. A., Walsh P. C. (1978). Male infertility. *Campbells Urology*.

[103] James S. A., Bilbiss L., Muhammad B. Y. (2007). The effects of *Catharanthus roseus* (L) G. Don 1838 aqueous leaf extract on some liver enzymes, serum proteins and vital organs. *Science World Journal*.

[104] Kuria KA, Muriuki G (1984). A new cardiotonic agent from Ajuga remota benth (Labiatae). *East African Medical Journal*.

[105] Gachathi F.N. (1993). *Kikuyu Botanical Dictionary of Plant Names and Uses*.

[106] Mueller M. S., Runyambo N., Wagner I., Borrmann S., Dietz K., Heide L. (2004). Randomized controlled trial of a traditional preparation of *Artemisia annua* L. (Annual Wormwood) in the treatment of malaria. *Transactions of the Royal Society of Tropical Medicine and Hygiene*.

[107] Blanke C. H., Naisabha G. B., Balema M. B., Mbaruku G. M., Heide L., Müller M. S. (2008). HerbaArtemisiae annuaetea preparation compared to sulfadoxine-pyrimethamine in the treatment of uncomplicated falciparum malaria in adults: a randomized double-blind clinical trial. *Tropical Doctor*.

[108] Barlow-Benschop N. M., Gamba C., Barlow S. P., Blasco T. M. The effect of a homeopathic neem preparation for the prophylaxis of malaria. An exploratory trial in an at-home setting in Tanzania. https://pdfs.semanticscholar.org/177a/390381a2a7c7a19566a20f57330f50751101.pdf.

[109] Bidla G., Titanji V. P. K., Jako B. (2004). Anti-plasmodial activity of seven plants used in African folk medicine. *Indian Journal of Pharmacy*.

[110] Ohigashi H., Huffman M. A., Izutsu D. (1994). Toward the chemical ecology of medicinal plant use in chimpanzees: the case of *Vernonia amygdalina*, a plant used by wild chimpanzees possibly for parasite-related diseases. *Journal of Chemical Ecology*.

[111] Challand S., Willcox M. (2009). A clinical trial of the traditional medicine *Vernonia amygdalina* in the treatment of uncomplicated malaria. *The Journal of Alternative and Complementary Medicine*.

[112] Bah S., Jäger A. K., Adsersen A., Diallo D., Paulsen B. S. (2007). Antiplasmodial and GABAA-benzodiazepine receptor binding activities of five plants used in traditional medicine in Mali, West Africa. *Journal of Ethnopharmacology*.

[113] Abdel-Azim N. S., Shams K. A., Shahat A. A. (2011). Egyptian herbal drug industry: challenges and future prospects. *Research Journal of Medicinal Plant*.

[114] Dold A. L., Cocks M. L. (2002). The trade in medicinal plants in the Eastern Cape province, South Africa. *South African Journal of Sciences*.

[115] The New Humanitarian, Small farmers cash in on Artemisinin production, http://www.thenewhumanitarian.org/report/82486/kenya-small-farmers-cash-artemisinin-production

[116] Oketch-Rabah H., Christensen S., Frydenvang K. (1998). Antiprotozoal properties of 16,17-Dihydroxybrachycalyxolide from *Vernonia brachycalyx*. *Planta Medica*.

[117] Ssegawa P., Kasenene J. M. (2007). Plants for malaria treatment in Southern Uganda: traditional use, preference and ecological viability. *Journal of Ethnobiology*.

[118] Bbosa S., Kyegombe D. B., Lubega A. (2013). Anti-plasmodium falciparum activity of Aloe dawei and Justicia betonica. *African Journal of Pharmacy and Pharmacology*.

[119] Perez H. A., De la Rosa M., Apitz R. (1994). In vivo activity of ajoene against rodent malaria. *Antimicrobial Agents and Chemotherapy*.

[120] Strangeland T., Alele P. E., Katuura E., Lye K. A. (2011). Plants used to treat malaria in Nyakayojo sub-county, Western Uganda. *Journal of Ethnopharmacology*.

[121] Kigondu E. V. M., Rukunga G. M., Keriko J. M. (2009). Anti-parasitic activity and cytotoxicity of selected medicinal plants from Kenya. *Journal of Ethnopharmacology*.

[122] Inbaneson S. J., Ravikumar S., Suganthi P. (2012). In vitro antiplasmodial effect of ethanolic extracts of coastal medicinal plants along Palk Strait against Plasmodium falciparum. *Asian Pacific Journal of Tropical Biomedicine*.

[123] Lemma M. T., Ahmed A. M., Elhady M. T. (2017). Medicinal plants for in vitro antiplasmodial activities: a systematic review of literature. *Parasitology International*.

[124] Yarnell E. (2014). Artemisia annua (sweet annie), other artemisia species, artemisinin, artemisinin derivatives, and malaria. *Journal of Restorative Medicine*.

[125] Katuura E., Waako P., Tabuti J. R. S., Bukenya-Ziraba R., Ogwal-Okeng J. (2007). Antiplasmodial activity of extracts of selected medicinal plants used by local communities in Western Uganda for treatment of malaria. *African Journal of Ecology*.

[126] Kabubbi Z. N., Mbaria J. M., Mbaabu M. (2015). Acute toxicity studies of Caranthus roseus aqueous extract in male Wistar rats. *African Journal of Pharmacology and Therapeutics*.

[127] Liu N. Q., Van Der Kooy F., Verpoorte R. (2009). Artemisia afra: a potential flagship for African medicinal plants?. *South African Journal of Botany*.

[128] Muganga R., Angenot L., Tits M., Frédérich M. (2010). Antiplasmodial and cytotoxic activities of Rwandan medicinal plants used in the treatment of malaria. *Journal of Ethnopharmacology*.

[129] Kohler I., Jenett-Siems K., Kraft C. (2002). Herbal remedies traditionally used against malaria in Ghana: bioassay-guided fractionation of Microglossa pyrifolia (Asteraceae). *Zeitschrift fur Naturforschung C*.

[130] Lacroix D., Prado S., Kamoga D. (2011). Antiplasmodial and cytotoxic activities of medicinal plants traditionally used in the village of Kiohima, Uganda. *Journal of Ethnopharmacology*.

[131] Onguén P. A., Ntie-Kang F., Lifongo L. L., Ndom J., Sippl W., Mbaze L. (2013). The potential of anti-malarial compounds derived from African medicinal plants. Part I: a pharmacological evaluation of alkaloids and terpenoids. *Malaria Journal*.

[132] Goffin E., Ziemons E., De Mol P. (2002). In vitro antiplasmodial activity of Tithonia diversifolia and identification of its main active constituent: tagitinin C. *Planta Medica*.

[133] Oyewole I. O., Ibidapo C. A., Moronkola D. O. (2008). Anti-malarial and repellent activities of Tithonia diversifolia (Hemsl.) leaf extracts. *Journal of Medicinal Plants Research*.

[134] Obbo C. J. D., Kariuki S. T., Gathirwa J. W., Olaho-Mukani W., Cheplogoi P. K., Mwangi E. M. (2019). In vitro antiplasmodial, antitrypanosomal and antileishmanial activities of selected medicinal plants from Ugandan flora: refocusing into multi-component potentials. *Journal of Ethnopharmacology*.

[135] Omoregie E. S., Pal A., Sisodia B. (2011). In vitro antimalarial and cytotoxic activities of leaf extracts of *Vernonia amygdalina* (Del.). *Nigerian Journal of Basic and Applied Sciences*.

[136] Lacroix D., Prado S., Deville A. (2009). Hydroperoxy-cycloartane triterpenoids from the leaves of Markhamia lutea, a plant ingested by wild chimpanzees. *Phytochemistry*.

[137] Ntie-Kang F., Onguene P. A., Lifongo L. L. (2014). The potential of anti-malarial compounds derived from African medicinal plants, Part II: a pharmacological evaluation of non-alkaloids and non-terpenoids. *Malaria Journal*.

[138] Rangasamy D., Asirvatham D., Muthusamy J. (2008). Preliminary phytochemical screening and antimalarial studies of Spathodea campanulatum P. Beauv leaf extracts. *Ethnobotany Leaflets*.

[139] Okello D., Komakech R., Matsabisa M. G., Kang Y.-M. (2018). A review on the botanical aspects, phytochemical contents and pharmacological activities of Warburgia ugandensis. *Journal of Medicinal Plants Research*.

[140] Wube A. A., Bucar F., Gibbons S., Asres K., Rattray L., Croft S. L. (2010). Antiprotozoal activity of drimane and coloratane sesquiterpenes towards Trypanosoma brucei rhodesiense and Plasmodium falciparum in vitro. *Phytotherapy Research*.

[141] Were P. S., Kinyanjui P., Gicheru M. M., Mwangi E., Ozwara H. S. (2010). Prophylactic and curative activities of extracts from Warburgia ugandensis Sprague (Canellaceae) and Zanthoxylum usambarense (Engl.) Kokwaro (Rutaceae) against Plasmodium knowlesi and Plasmodium berghei. *Journal of Ethnopharmacology*.

[142] Melariri P., Campbell W., Etusim P., Smith P. (2011). Antiplasmodial properties and bioassay-guided fractionation of ethyl acetate extracts from Carica papaya leaves. *Journal of Parasitology Research*.

[143] Teng W.-C., Chan W., Suwanarusk R. (2019). In vitro antimalarial evaluations and cytotoxicity investigations of Carica papaya leaves and carpaine. *Natural Product Communications*.

[144] Nanyingi M. O., Kipsengeret K. B., Wagate C. G., Langat B. K., Asaava L. L., Midiwo J. O., Midiwo J. O., Clough J. (2010). In vitro and in vivo antiplasmodial activity of Kenyan medicinal plants. *Aspects of African Biodiversity: Proceedings of the Pan-Africa Chemistry Network*.

[145] Malebo H. M., Wiketye V., Katani S. J. (2015). In vivo antiplasmodial and toxicological effect of Maytenus senegalensis traditionally used in the treatment of malaria in Tanzania. *Malaria Journal*.

[146] Asres K., Bucar F., Knauder E., Yardley V., Kendrick H., Croft S. L. (2001). In vitro antiprotozoal activity of extract and compounds from the stem bark of Combretum molle. *Phytotherapy Research*.

[147] Froelich S., Onegi B., Kakooko A., Siems K., Schubert C., Jenett-Siems K. (2007). Plants traditionally used against malaria: phytochemical and pharmacological investigation of Momordica foetida. *Revista Brasileira de Farmacognosia*.

[148] Singh S. V., Manhas A., Kumar Y. (2017). Antimalarial activity and safety assessment of Flueggea virosa leaves and its major constituent with special emphasis on their mode of action. *Biomedicine and Pharmacotherapy*.

[149] Kaou A. M., Mahiou-Leddet V., Hutter S. (2008). Antimalarial activity of crude extracts from nine African medicinal plants. *Journal of Ethnopharmacology*.

[150] Bero J., Frédérich M., Quetin-Leclercq J. (2009). Antimalarial compounds isolated from plants used in traditional medicine. *Journal of Pharmacy and Pharmacology*.

[151] Memvanga P. B., Tona G. L., Mesia G. K., Lusakibanza M. M., Cimanga R. K. (2015). Antimalarial activity of medicinal plants from the Democratic Republic of Congo: a review. *Journal of Ethnopharmacology*.

[152] Iwalewa E. O., Suleiman M. M., Mdee L. K., Eloff J. N. (2009). Antifungal and antibacterial activities of different extracts ofHarungana madagascariensisstem bark. *Pharmaceutical Biology*.

[153] Iwalewa E. O., Omisore N. O., Adewunmi C. O. (2008). Anti-protozoan activities of Harungana madagascariensis stem bark extract on trichomonads and malaria. *Journal of Ethnopharmacology*.

[154] Deressa T., Mekonnen Y., Animut A. (2010). In vivo anti-malarial activities of Clerodendrum myricoides, Dodonea angustifolia and Aloe debrana against Plasmodium berghei. *Ethiopian Journal of Health and Development*.

[155] Smolenski S. J., Silinis H., Farnsworth N. R. (1975). Alkaloid screening VIII. *Lloydia*.

[156] Murugan K., Aarthi N., Kovendan K. (2015). Mosquitocidal and antiplasmodial activity of Senna occidentalis (Cassiae) and Ocimum basilicum (Lamiaceae) from Maruthamalai hills against Anopheles stephensi and Plasmodium falciparum. *Parasitology Research*.

[157] Muthaura C. N., Keriko J. M., Mutai C. (2015). Antiplasmodial potential of traditional antimalarial phytotherapy remedies used by the kwale community of the Kenyan Coast. *Journal of Ethnopharmacology*.

[158] Asase A., Akwetey G. A., Achel D. G. (2010). Ethnopharmacological use of herbal remedies for the treatment of malaria in the Dangme West district of Ghana. *Journal of Ethnopharmacology*.

[159] Khalid S. A., Duddeck H., Gonzalez-Sierra M. (1989). Isolation and characterization of an antimalarial agent of the neem tree Azadirachta indica. *Journal of Natural Products*.

[160] Bray D. H., Warhurst D. C., Connolly J. D., O’Neill M. J., Phillipson J. D. (1990). Plants as sources of antimalarial drugs. Part 7. Activity of some species of meliaceae plants and their constituent limonoids. *Phytotherapy Research*.

[161] Batista R., De Jesus Silva Júnior A., de Oliveira A. (2009). Plant-derived antimalarial agents: new leads and efficient phytomedicines. Part II: non-alkaloidal natural products. *Molecules*.

[162] Lusakibanza M., Mesia G., Tona G. (2010). In vitro and in vivo antimalarial and cytotoxic activity of five plants used in Congolese traditional medicine. *Journal of Ethnopharmacology*.

[163] Falade M. O., Akinboye D.O., Gbotosho G. O. (2014). In vitro and in vivo antimalarial activity of *Ficus thonningii* Blume (Moraceae) and *Lophira alata* banks (Ochnaceae), identified from the ethnomedicine of the Nigerian Middle Belt. *Journal of Parasitology Research*.

[164] Alli L., Adesokan A., Salawu A. (2016). Antimalarial activity of fractions of aqueous extract of Acacia nilotica root. *Journal of Intercultural Ethnopharmacology*.

[165] Ndjakou Lenta B., Vonthron-Sénécheau C., Fongang Soh R. (2007). In vitro antiprotozoal activities and cytotoxicity of some selected Cameroonian medicinal plants. *Journal of Ethnopharmacology*.

[166] Okpo S. O., Igwealor C. O., Eze G. I. (2016). Sub-acute toxicity study on the aqueous extract of Albizia zygia stem bark. *Journal of Pharmacy & Bioresources*.

[167] Katuura E., Waako P., Ogwal-Okeng J., Bukenya-Ziraba R. (2007). Traditional treatment of malaria in Mbarara district, Western Uganda. *African Journal of Ecology*.

[168] Pillay P., Maharaj V. J., Smith P. J. (2008). Investigating South African plants as a source of new antimalarial drugs. *Journal of Ethnopharmacology*.

[169] Endale M., Alao J., Akala H. (2012). Antiplasmodial Quinones from *Pentas longiflora* and *Pentas lanceolata*. *Planta Medica*.

[170] Chinwuba P., Akah P. A., Iiodigwe E. E. (2015). In vivo antiplasmodial activity of the ethanol stem extract and fractions of Citrus sinensis in mice. *Merit Research Journal of Medicine and Medical Sciences*.

[171] Burkill H. R. M. (2000). *The useful plants of West tropical Africa*.

[172] Nyasse B, Nono J, Sonke B, Denier C, Fontaine C (2004). Trypanocidal activity of bergenin, the major constituent of Flueggea virosa, on Trypanosoma brucei. *Die Pharmazie*.

[173] Gan L.-S., Fan C.-Q., Yang S.-P. (2006). Flueggenines A and B, two novel C,C-linked dimeric indolizidine alkaloids from *Flueggea virosa*. *Organic Letters*.

[174] Al-Rehaily A. J., Yousaf M., Ahmad M. S. (2015). Chemical and biological study of *Flueggea virosa* native to Saudi Arabia. *Chemistry of Natural Compounds*.

[175] Ajaiyoba E., Ashidi J., Abiodun O. (2005). Antimalarial ethnobotany: in vitro antiplasmodial activity of seven plants identified in the Nigerian middle belt. *Pharmaceutical Biology*.

[176] Muganga R., Angenot L., Tits M., Frédérich M. (2014). In vitro and in vivo antiplasmodial activity of three Rwandan medicinal plants and identification of their active compounds. *Planta Medica*.

[177] Asnake S., Teklehaymanot T., Hymete A., Erko B., Giday M. (2015). Evaluation of the antiplasmodial properties of selected plants in southern Ethiopia. *BMC Complementary and Alternative Medicine*.

[178] Rukunga G. M., Muregi F. W., Tolo F. M. The antiplasmodial activity of spermine alkaloids isolated from Albizia gummifera. *Fitoterapia*.

[179] Seo Y., Berger J. M., Hoch J. (2002). A New Triterpene Saponin fromPittosporumviridiflorumfrom the Madagascar Rainforest1. *Journal of Natural Products*.

[180] Nyongbela K. D., Lannang A. M., Ayimele G. A., Ngemenya M. N., Bickle Q., Efange S. (2013). Isolation and identification of an antiparasitic triterpenoid estersaponin from the stem bark of Pittosporum mannii (Pittosporaceae). *Asian Pacific Journal of Tropical Disease*.

[181] Nkunya M. H. H., Jonker S. A., De Gelder R., Wachira S. W., Kihampa C. (2004). (±)-Schefflone: a trimeric monoterpenoid from the root bark of *Uvaria scheffleri*. *Phytochemistry*.

[182] Nkunya M., Weenen H., Bray D., Mgani Q., Mwasumbi L. (1991). Antimalarial activity of Tanzanian plants and their active constituents: The GenusUvaria. *Planta Medica*.

[183] Iwu M., Court W. (1979). Alkaloids of Rauwolfia mombasiana stem bark. *Planta Medica*.

[184] Weenen H., Nkunya M., Bray D., Mwasumbi L., Kinabo L., Kilimali V. (1990). Antimalarial activity of Tanzanian medicinal plants. *Planta Medica*.

[185] Akbar E., Malik A., Afza N., Hai S. M. A. (2002). Flavone glycosides and bergenin derivatives from Tridax procumbens. *Heterocycles*.

[186] Holeman M., Theron E., Pinel R. (1994). Centella asiatica analyses by GC-MS and infrared MS. *Parfums Cosmetica Aromes*.

[187] Kayembe J. S., Taba K. M., Ntumba K., Tshiongo M. T. C., Kazadi T. K. (2010). In vitro antimalarial activity of 20 quinones isolated from four plants used by traditional healers in the Democratic Republic of Congo. *Journal of Medicinal Plants Research*.

[188] Tona L., Mesia K., Ngimbi N. P. (2001). In-vivo antimalarial activity of *Cassia occidentalism Morinda morindoides* and *Phyllanthus niruri*. *Annals of Tropical Medicine & Parasitology*.

